# An orally administered biohybrid complex leveraging probiotic guidance to treat colorectal cancer via *in situ* vaccination

**DOI:** 10.1016/j.mtbio.2026.103445

**Published:** 2026-07-14

**Authors:** Wenfei Fan, Shuaiguang Li, Xue Wang, Jingya Xu, Shan Jiang, Haixia Shen, Yanran Yue, Zhonghua Dong, Xuan Wang, Haiping Hu, Wei Xu

**Affiliations:** a Department of Clinical Pharmacy, The First Affiliated Hospital of Shandong First Medical University & Shandong Provincial Qianfoshan Hospital, Jinan, Shandong, 250014, China; b State Key Laboratory of Advanced Drug Delivery and Release Systems, Shandong Luye Pharmaceutical Co., Ltd., Yantai, Shandong, 264003, China; c School of Pharmaceutical Sciences, Shandong University of Traditional Chinese Medicine, Jinan, Shandong, 250355, China; d School of Pharmacy, The Shandong Second Medical University, Weifang, Shandong, 261053, China; e Department of Pharmacy, The Second Affiliated Hospital of Shandong First Medical, University, Tai'an, Shandong, 271000, China

**Keywords:** Colorectal cancer, Probiotic-guided delivery, Chemo-immunotherapy, Tumor immune microenvironment, *In situ* vaccine

## Abstract

Oral therapy for colorectal cancer (CRC) holds inherent promise for enhancing patient compliance and enabling gastrointestinal targeting, while its application is hampered by low oral drug utilization efficiency and precise colonic localization. This study developes an orally delivered biohybrid complex, CS@CB-Lipo@5-FU/R837, which leverages the hypoxic tropism of the probiotic *Clostridium butyricum* (CB) for active tumor targeting. The core of the system consists of nanoliposomes co-loaded with 5-fluorouracil (5-FU) and the TLR7 agonist, R837, which are site-specifically anchored to CB via bioorthogonal conjugation. A chitosan-based outer coating ensures gastrointestinal stability and enables enzyme-responsive drug release in the tumor-colonized environment. Within the tumor, the complex orchestrates a coordinated immunotherapeutic cascade: 5-FU induces immunogenic cell death, releasing tumor antigens and damage-associated molecular patterns, while R837 promotes dendritic cell maturation and antigen presentation. This spatiotemporally coupled “antigen-adjuvant' delivery effectively mimics an *in situ* vaccination mechanism, stimulating potent antitumor immunity. Evaluation in orthotopic and subcutaneous CRC models shows treatment leads to tumor growth inhibition, remodeling of the immune microenvironment, and extended survival. This work establishes a versatile strategy for oral *in situ* vaccines based on probiotic-guided delivery and localized immune activation.

## Highlights

• An oral biohybrid complex is engineered for colorectal cancer therapy.
• Chitosan coating enables colon-specific enzyme-responsive drug release.
• Probiotic acts as a living vehicle for targeted tumor accumulation.
• Spatiotemporally coupled chemo-immunotherapy mimics *in situ* vaccination.

## Graphical abstract

**Figure:**
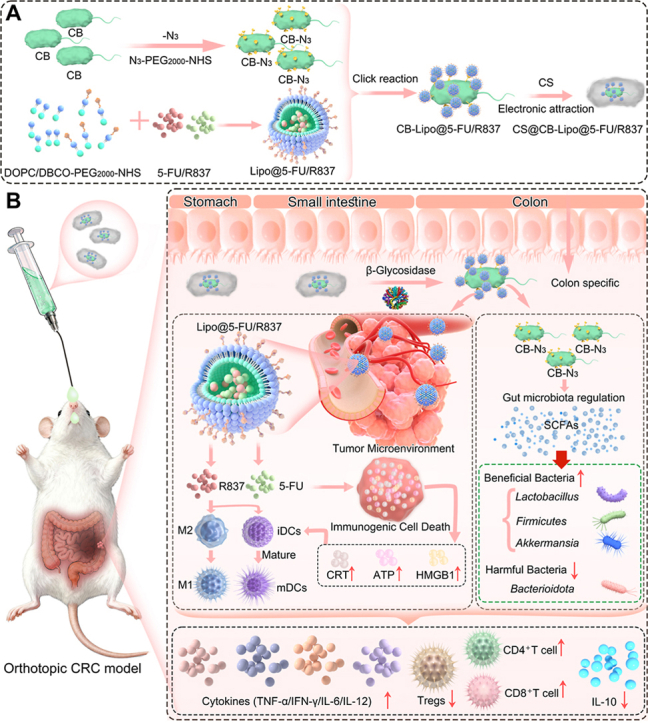

## 1 Introduction

Colorectal cancer (CRC) remains a leading cause of cancer-related mortality worldwide, underscoring the urgent need for more effective therapeutic strategies [1,2]. Conventional chemotherapy, while cytotoxic, often fails due to dose-limiting systemic toxicity, drug resistance and inadequate tumor-specific accumulation [3]. This ongoing challenge of non-specific drug distribution implies the critical demand for innovative delivery platforms that can enhance drug targeting to tumors, enhance tumor-specific drug accumulation and minimize off-target effect [4,5]. Given the complex pathogenesis of CRC, single-modality therapies often demonstrate limited effectiveness, prompting a shift toward combined treatment strategies. Among these, integrated “chemo-immunotherapy' has emerged as a promising approach for the treatment of CRC [6,7].

The delivery challenges confronting conventional chemotherapy are well exemplified by 5-fluorouracil (5-FU), a first-line agent in CRC management [8]. Despite its widespread use, 5-FU faces significant limitations: poor oral bioavailability, highly variable pharmacokinetics, and insufficient tumor accumulation even following intravenous administration [9]. Consequently, enhancing the local concentration of 5-FU in CRC tissues represents a significant strategy for improving therapeutic outcomes [10,11]. The efficacy of 5-FU in targeting proliferating tumor cells is often compromised by its concomitant inhibition of immune cells in the tumor microenvironment, which can foster an immunosuppressive state [12]. Against this backdrop, the advantages of the chemo-immunotherapy combination strategy have become prominent: by introducing immune agonists to counteract the inhibitory effects of chemotherapy and synergistically enhance anti-tumor immunity [6,13]. Various immune adjuvants have been employed in tumor immunotherapy [14]. The signaling pathway targeting Toll-like receptors (TLR) has been confirmed to be the key to effectively activating the innate immune system [15]. Miquimod (R837), a TLR7 agonist with well-tolerated safety approved by the FDA, are highly hydrophobic and its target is on the inner membrane of endosomes or lysosomes [16]. The systemic administration of R837 may induce hyperimmunity or off-target effects [17]. Therefore, strategies for spatially controlled R837 release within the tumor microenvironment is necessitated.

Oral administration represents an ideal route for CRC treatment, leveraging the physiological connection to achieve natural targeting while significantly improving patient compliance [18,19]. As a result, it has emerged as a significant area of research in CRC therapy. Nanotechnology is increasingly influential in cancer treatment, with liposomes being a prominent nanocarrier due to their superior drug loading capacity, favorable biocompatibility, and well-established safety profile [20]. At present, extensive research efforts have been directed towards the development of oral delivery system [21], such as microsphere sponge-based tablets, polysaccharide-based soft nano-in-micro agglomerates [22], and crystalline polymer microparticles, to enhance drug accumulation in the colorectal cavity [23]. However, the development of oral nanocarriers for CRC has been stymied by a major hurdle: most oral nanoparticle formulations are released or absorbed prematurely in the upper gastrointestinal tract, making it difficult for effective drugs to reach the local colorectal tumor. This not only severely limits drug accessibility at the target lesion but also makes it difficult to induce a potent local anti-tumor immune response.

Probiotics have recently emerged as a promising class of biological drug carriers for oral cancer therapy due to their excellent safety profiles [24,25], inherent resistance to harsh gastrointestinal conditions, and natural colonization ability within the gut [26]. Moreover, many probiotic strains possess intrinsic tumor-homing capabilities, particularly toward hypoxic and inflamed regions characteristic of CRC lesions [27,28]. Among the commonly studied strains, including *Lactobacillus reuteri* [29], *Lactobacillus acidophilus* [30], and *Clostridium butyricum* [31,32], the latter has demonstrated a robust capacity to selectively accumulate within hypoxic tumor microenvironments owing to its anaerobic nature. These attributes highlight *Clostridium butyricum* (CB, ATCC 19398) as an attractive “biological navigation vector' for precise CRC targeting following oral administration. However, despite their promise, probiotic-based carriers may still encounter survival barriers in the upper gastrointestinal tract prior to reaching the colorectal tumor site [33]. To enhance their viability during gastrointestinal passage, bioadhesive materials such as chitosan (CS) [34] and alginate [35] have been widely employed to construct protective coatings on probiotic delivery systems, significantly improving stability and colon-targeted accumulation.

**Scheme 1:**
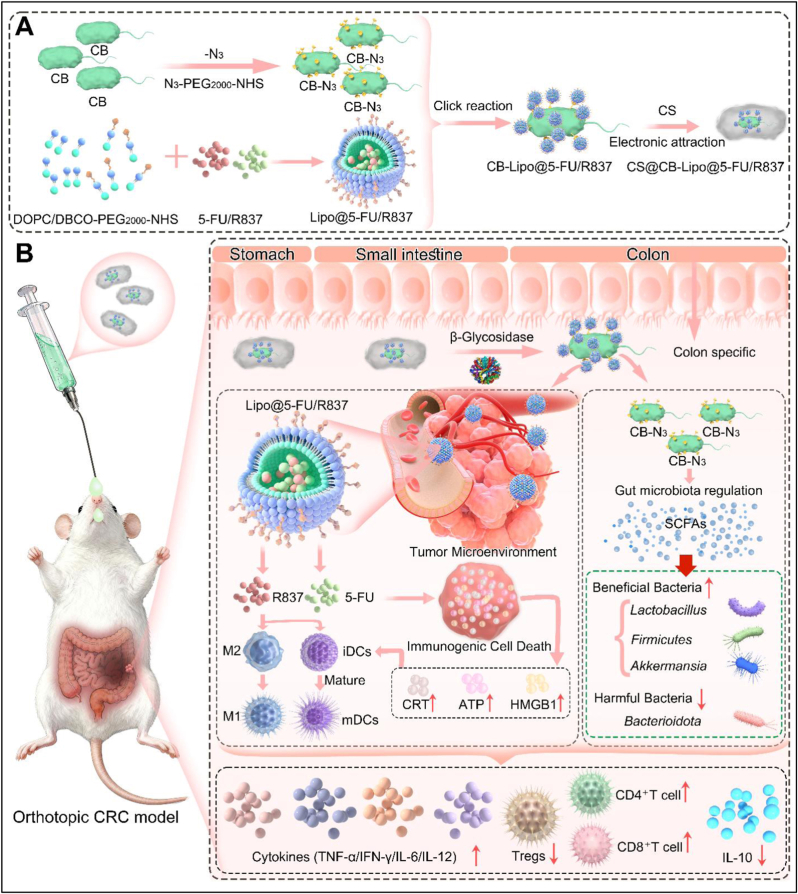
**Schematic diagram of the mechanism of CS@CB-Lipo@5-FU/R837 of orthotopic CRC therapy.** (A) Synthesis pathway of CS@CB-Lipo@5-FU/R837 complexes, DBCO-modified liposomes co-loaded with 5-FU and R837 were conjugated to azide-functionalized *Clostridium butyricum* (CB) via bioorthogonal click chemistry, followed by coating with a chitosan (CS) shell. (B) Upon oral administration, the CS layer protects the system through the gastrointestinal tract and is degraded by β-glucosidase in the colon, releasing CB-Lipo@5-FU/R837. The probiotic carrier then actively targets hypoxic tumor regions, where the released 5-FU induces ICD, exposing tumor antigens and DAMPs, while R837 activates dendritic cells and promotes M1 macrophage polarization. Together, these effects initiate an *in situ* vaccination response, enhancing T cell infiltration and antitumor immunity.

This study presents an orally administered complex (CS@CB-Lipo@5-FU/R837), engineered as a cascade-reaction platform for CRC treatment. The system sequentially accomplishes (Scheme 1): (1) enzymatic activation and probiotic-guided tumor targeting, followed by (2) localized induction of immunogenic cell death (ICD) coupled with adjuvant release within the tumor microenvironment (TME). The spatiotemporally controlled “antigen-release-immune-activation' cycle functions as an *in situ* vaccination mechanism, thereby producing marked anti-tumor effects and survival improvement in murine models. These findings offer a transformative strategy for oral chemo-immunotherapy by leveraging probiotic-guided targeted delivery.

## 2 Materials and methods

### 2.1 Chemicals and materials

5-Fluorouracil (5-FU, Cat# F6173), imiquimod hydrochloride (R837·HCl, Cat# I911660) and chitosan (degree of deacetylation 80%, MW = 500,000 Da, Cat# C915934) were purchased from Macklin Biochemical Co., Ltd (Shanghai, China). 1,2-Dioleoyl-sn-glycero-3-phosphocholine (DOPC, ≥99.0%, Cat# V30082) and β-glucosidase (100 U g^−1^, Cat# S24786) were purchased from Shanghai Yuanye Bio-Technology Co., Ltd (Shanghai, China). Dibenzocyclooctyne-poly (ethylene glycol) 2000-N-hydroxysuccinimide (DCBO-PEG_2000_-NHS, 98%), azido-poly (ethylene glycol) 2000-N-hydroxysuccinimide (N_3_-PEG_2000_-NHS, 95%), and azido-poly (ethylene glycol) 4-N-hydroxysuccinimide (N_3_-PEG_4_-NHS, 97%) were acquired from Weihua Biochemical Co., Ltd (Guangzhou, China). ActivAb™ Streptavidin/Solar Fluor 488 (AF488, 1 mg mL-1), dibenzocyclooctyne-AF488 (DBCO-AF488), and 4′,6-diamidino-2-phenylindole (DAPI, 1 mg mL-1) were purchased from Solarbio Co., Ltd. (Shanghai, China). RPMI-1640 medium, Dulbecco's Modified Eagle Medium (DMEM), fetal bovine serum (FBS), penicillin-streptomycin (double antibody), phosphate-buffered saline (PBS), and trypsin-ethylenediaminetetraacetic acid (trypsin-EDTA, 0.05%) were obtained from Gibco (Pittsburgh, USA). *Clostridium butyricum* (CB, ATCC 19398) was purchased from Donghai Pharmaceutical Co., Ltd (Qingdao, China). The Annexin V-FITC/PI Apoptosis Detection Kit (Cat# C1062S) was acquired from Beyotime Biotechnology Co., Ltd (Shanghai, China). GAPDH Mouse Monoclonal Antibody (5-E10, Cat# EM1101), HMGB1 rabbit monoclonal antibody (Cat# ET1601-2) and Calreticulin (CRT) rabbit monoclonal antibody (Cat# ET1608-60) were purchased from Hua'an Biotechnology Co., Ltd (Hangzhou, China). Mouse ELISA Kits (GZMB, IL-6, TNF–α, IL-10, and IL-12) were obtained from Boster Biological Technology Co., Ltd (Wuhan, China). Anaerobic gas-generating packs and anaerobic bags were purchased from Haibo Biotechnology Co., Ltd (Qingdao, China). Macrophage colony-stimulating factor (M-CSF, Cat# AF31502), granulocyte-macrophage colony-stimulating factor (GM-CSF, Cat# 31503), IL-13 (Cat# 21013) and IL-4 (Cat# 21414) were acquired from PeproTech, Inc (USA). D-Luciferin (Cat# C3654) was manufactured by APExBIO Technology Inc (USA). Antibodies for flow cytometry were purchased from Bioleng Biotech Co., Ltd (USA). and BD Medical Devices Co., Ltd (Shanghai, China).

### 2.2 Synthesis of Lipo@5-FU/R837 and characterization

Hydrophilic drugs 5-fluorouracil (5-FU) and imiquimod hydrochloride (R837·HCl) were encapsulated into nanoliposomes using the thin-film hydration method. Precisely weighed lipid components, including DOPC and DSPE-PEG_2000_-DBCO, were mixed at a defined molar ratio (DBCO-PEG_2000_-NHS: 10.5 mg; DOPC: 8.5 mg) and dissolved in 1 mL of anhydrous ethanol to form the organic phase.

Separately, 3 mg of 5-FU and R837·HCl were dissolved in 10 mL of deionized water to prepare the aqueous phase. The aqueous solution was gradually added dropwise into the organic phase under gentle water-bath sonication until a homogeneous water-in-oil (W/O) emulsion was obtained. Subsequently, the organic phase was injected into the aqueous phase at a rate of 1 mL min-1 under continuous stirring (500 rpm). The mixture was stirred overnight to ensure complete evaporation of ethanol. The resulting liposomes were purified using ultrafiltration tubes (molecular weight cutoff: 10,000 Da) and centrifuged at 10,000 rpm for 30 min to remove unencapsulated drugs, yielding drug-loaded nanoliposomes (Lipo@5-FU/R837).

The morphology of the liposomes was observed by transmission electron microscopy (TEM, JEM-2100F, JEOL, Japan). The particle size and zeta potential were determined using a dynamic light scattering (DLS, NS-90Z Plus, Omec, China) analyzer. The amount of unencapsulated drug in the filtrate was quantified by high-performance liquid chromatography (HPLC, 1260 Infinity III Prime, Japan) to calculate the encapsulation efficiency (EE%) and drug loading capacity (DL%) of Lipo@5-FU/R837.

### 2.3 Synthesis and characterization of CS@CB-Lipo@5-FU/R837

After large-scale anaerobic cultivation, CB colonies were collected. For surface azide functionalization, the bacterial suspension (10^8^ CFU mL^−1^) was reacted with 50 μM N_3_-PEG_4_-NHS or N_3_-PEG_2000_-NHS (dissolved in DMSO) in a total reaction volume of 1 mL at 37 °C under shaking (220 rpm) for 2-4 h [36,37]. The mixture was centrifuged (3000 rpm, 5 min), and the azide-functionalized bacteria (CB-N_3_) were resuspended in sterile PBS containing 1%DMSO and washed twice by centrifugation. Then, we used a UV-vis spectrophotometer (UV-9000, METASH, Shanghai) by measuring absorbance to test the labeling of the azide group.

For bioorthogonal conjugation, CB-N_3_ (10^8^ CFU) was incubated with Lipo@5-FU/R837 (1 mg mL^−1^) at a 1:1 vol ratio at 37 °C under gentle shaking (100 rpm) for 4 h. The azide groups on the probiotic surface reacted specifically with the DBCO moieties on the liposomes, forming stable CB-Lipo@5-FU/R837 conjugates via strain-promoted azide-alkyne cycloaddition (SPAAC), forming stable CB-Lipo@5-FU/R837 conjugates.

After incubation, the conjugates were allowed to settle overnight at 4 °C and collected by centrifugation (3000 rpm, 5 min, 4 °C). The resulting pellet containing CB-Lipo@5-FU/R837 was resuspended to a final concentration of 10^8^ CFU mL^−1^ or used directly for chitosan coating.

Drug concentrations in the supernatant were determined by HPLC after demulsification, and the loading efficiencies of 5-FU and R837 were calculated as follows:

Drug loading efficiency (5-FU) = 5-FU content/Total colonies of CB (μg CFU^−1^)

Drug loading efficiency (R837) = R837 content/Total colonies of CB (μg/CFU^−1^)

To obtain the CS-coated composite, the CB-Lipo@5-FU/R837 suspension (10^8^ CFU mL^−1^) was mixed with 1% (v/v) CS solution (3 mg mL^−1^, dissolved in 1% acetic acid, pH = 5, MW = 500,000 Da). The mixture was stirred magnetically at 100 rpm for 2 h at room temperature to facilitate electrostatic adsorption of chitosan onto the probiotic-liposome complex. Afterward, the mixture was centrifuged (3000 rpm, 5 min), and the precipitate was collected to yield the final CS-coated composite, denoted as CS@CB-Lipo@5-FU/R837. Furthermore, the chemical functional groups of CS@CB-Lipo@5-FU/R837 were detected by using TEM and FT-IR (VERTEX 70, Bruker, Germany).

### 2.4 *In vitro* stability of CS@CB-Lipo@5-FU/R837

CB, CB-Lipo@5-FU/R837, and CS@CB-Lipo@5-FU/R837 suspensions were serially diluted to equivalent concentrations and plated for optical density (OD_600_) measurements to assess probiotic viability and stability. To verify the *in vitro* serum stability of Lipo@5-FU/R837 and CS@CB-Lipo@5-FU/R837, Lipo@5-FU/R837 and CS@CB-Lipo@5-FU/R837 were dispersed in sterile PBS (pH 7.4) containing 10% (v/v) fetal bovine serum and incubated at 37 °C in the dark respectively. Samples were taken at 0, 6, 12, 24 and 48 h, diluted with ultrapure water appropriately, and the hydrodynamic diameter and Zeta potential were measured using a dynamic light scattering instrument. Each sample was measured three times. In addition, the serum blank control with PBS instead of nanoparticles was used in the same way to eliminate the interference of serum particles. Take an appropriate amount of incubation solution at 12 h, collect Lipo@5-FU/R837 and CS@CB-Lipo@5-FU/R837, wash twice with ultrapure water, resuspend, drop onto carbon support membrane copper mesh, negatively dye with 2% sodium phosphotungstate, naturally dry and place under transmission electron microscope to observe morphology and dispersion state.

### 2.5 *In vitro* release study

The *in vitro* release behavior of CS@CB-Lipo@5-FU was evaluated under simulated gastrointestinal conditions. The composite was incubated at 37 °C under anaerobic conditions in simulated gastric fluid (SGF, pH = 1.2), simulated intestinal fluid (SIF, pH = 6.8), and simulated colonic fluid (SCF, pH = 7.4, with or without β-glucosidase, 5 U mL^−1^). Incubation was performed for 2 h in SGF, 4 h in SIF, and up to 24 h in SCF.

At predetermined time intervals, 0.5 mL of the release medium was withdrawn and replaced with an equal volume of fresh corresponding medium to maintain sink conditions. Samples were centrifuged (4500 rpm, 5 min), and the supernatants were mixed with two-fold volumes of methanol to disrupt the emulsion. The concentration of released 5-FU was quantified by HPLC. The cumulative release percentage was calculated according to the equation:

Released liposomes (%) = 5-FU content in upper suspension/5-FU content in CS@CB-Lipo@5-FU/R837 × 100

### 2.6 *In vitro* cytotoxicity by CCK8 assay

CT26 (Cat# CL0863, Fenghui Biotechnology, China), MC38 (Cat# CBPG0089, COBIOER, China), and L929 (Cat# CBP60878, COBIOER, China) cells were seeded in 96-well plates at a density of 5 × 10^3^ cells per well and cultured overnight at 37 °C in a humidified atmosphere containing 5% CO_2_. The next day, the culture medium was replaced with serum-free medium, and cells were divided into three treatment groups: 5-FU, Lipo@5-FU, and Lipo@5-FU/R837. The formulations were added at various drug concentrations and incubated for 24 or 48 h. Subsequently, 10% CCK-8 reagent was added to each well and incubated according to the manufacturer's instructions. Plates were incubated at 37 °C for 1 h, and absorbance at 450 nm was measured using a microplate reader (Varioskan LUX, Thermo Fisher Scientific, USA).

### 2.7 Annexin V-FITC/PI assay and calcein-AM/PI double stain

CT26 cells were seeded into 6-well plates at a density of 3 × 10^5^ cells per well and treated with one of 4 formulations: Control (PBS), 5-FU, Lipo@5-FU, Lipo@5-FU/R837. Cell apoptosis was quantified using an Annexin V-FITC/PI apoptosis detection kit (Cat# C1062S, Beyotime, China) following the manufacturer's protocol, and apoptotic ratios were analyzed by flow cytometry (FCM, CytoFELX, Beckman, USA). For live/dead staining, cells were incubated with the same formulations for 24 h, followed by staining with a Calcein-AM/PI kit (Cat# C2015S, Beyotime, China). Fluorescence images were captured using a fluorescence microscope (Ixplore IX85, Olympus, Japan) to visualize viable (green) and dead (red) cells.

### 2.8 Cellular uptake and lysosomal escape

To evaluate cellular uptake, CT26 cells were incubated with Alexa Fluor 488-labeled nanoparticles (CS@Lipo@AF488). Cells (2 × 10^5^ cells per well) were seeded in 6-well plates and allowed to adhere overnight. Uptake efficiency was analyzed using confocal laser scanning microscopy (CLSM, Zeiss Cell Discoverer 7, Germany) and FCM. For lysosomal escape observation, CT26 cells were treated similarly for 1, 2, and 4 h, then stained with Lyso-Tracker Red (50 nM, Cat# C1046, Beyotime, China) to label lysosomes and Hoechst 33342 (1 mg mL^−1^, Cat# C0021, Solarbio, China) to stain nuclei. The intracellular distribution of nanoparticles was imaged using CLSM to assess endolysosomal escape.

### 2.9 Validation of ICD effects *in vitro*

To evaluate the ability of different formulations to induce ICD, this study examined the characteristic damage-related molecular patterns (DAMPs) associated with ICD. CT26 cells were seeded in 6-well plates at a density of 2 × 10^5^ cells per well. After overnight attachment, CT26 cells were incubated for 24 h over different drugs. The cells were collected and the exposure of CRT on the cell membrane surface and the release of extracellular HMGB1 were detected by immunofluorescence staining. The fluorescence profiles of CRT (green fluorescence) and HMGB1 (green fluorescence) were observed and recorded by CLSM. At the same time, the cell culture supernatants of each group were collected, and the ATP content was determined according to the instructions of the ATP detection kit to reflect the ATP level released by cell lysis. Additionally, total cell proteins were collected, and the protein expression levels of CRT and HMGB1 were detected by Western blot (WB), and semi-quantitative analysis of gray values was performed to compare the expression differences of ICD markers among the treatment groups. These methods comprehensively verified the intensity of ICD induction by different nanomaterial formulations from multiple dimensions including protein localization, secretion level, and release amount.

### 2.10 *In vitro* induction of BMDMs repolarization

Bone marrow-derived macrophages (BMDMs) were isolated from BALB/c mice. Mice were euthanized by cervical dislocation, and femurs and tibias were aseptically harvested. After removing residual muscle tissue, bones were sterilized in 75% ethanol for 1 min and washed twice with PBS. The marrow cavities were flushed with sterile PBS using a 10 mL syringe until clear. The collected bone marrow cells were filtered through a 70 μm cell strainer and centrifuged. Red blood cells were lysed with 3 mL of lysis buffer for 5 min, and the remaining cells were resuspended in complete RPMI 1640 medium supplemented with 10% FBS, 1% penicillin-streptomycin, and 20 ng mL^−1^ M-CSF (Cat# AF31502, USA). MycoClear-3 (Cat# C3472-0100, Viva Cell, Germany) was added to the primary cells (BMDMs) extracted above. Cells were seeded in 6-well plates at a density of 2 × 10^6^ cells per well and cultured for 6 days for macrophage differentiation. On day 6, BMDMs were pretreated with 20 ng mL^−1^ IL-4 and IL-13 to induce M2 polarization. Subsequently, cells were treated with 5-FU, CB-Lipo@5-FU/R837, or CS@CB-Lipo@5-FU/R837 for 24 h. The expression levels of CD206 and CD86 on F4/80^+^ macrophages were measured by FCM to assess macrophage repolarization.

### 2.11 *In vitro* induction of BMDCs maturation

CT26 cells were seeded into 6-well plates at a density of 3 × 10^5^ cells per well and cultured overnight in 1 mL of complete RPMI 1640 medium. MycoClear-3 was added to the primary cells (BMDCs) extracted above. The cells were then treated with different formulations (LPS, CB-Lipo@5-FU/R837, CS@CB-Lipo@5-FU/R837) for 24 h. The supernatant was collected and centrifuged to remove cellular debris. Bone marrow-derived dendritic cells (BMDCs) were prepared as previously described by BALB/c mice [38]. On day 6 of differentiation, BMDCs were exposed to the collected CT26-conditioned media (30% v/v of the total culture volume) from each treatment group for 24 h. On day 7, both suspended and semi-adherent BMDCs were harvested, and the expression of CD80 and CD86 on CD11c^+^ cells was analyzed by FCM to assess BMDCs maturation.

### 2.12 Establishment of orthotopic and subcutaneous CRC models

All animal experiments were conducted in accordance with the guidelines approved by the Animal Ethics Committees of Shandong First Medical University and Shandong Qianfoshan Hospital (Approval ID. 2024111203). Female BALB/c and male C57BL/6 mice (6-7 weeks old, weighing 16-24 g) were supplied by Beijing Vital River Laboratory Animal Technology Co., Ltd. (Beijing, China; License Nos. SCXK (Jing) 2021-0006). After one week of acclimation under specific pathogen-free (SPF) conditions, the mice were randomly allocated into seven experimental groups. Mice were anesthetized with 3% isoflurane maintained on 1.5% isoflurane in oxygen, a small abdominal incision was made to expose the cecum and distal colon. For the orthotopic colorectal cancer model, CT26-luc (Cat# CBP30258L, COBIOER, China) cells (5 × 10^5^ cells) were injected into the cecal wall. The abdominal incision and skin were then sutured sequentially. At designated time points, D-luciferin (150 mg kg^−1^) was administered intraperitoneally, and bioluminescence in the abdominal region was monitored using an In Vivo Imaging System (IVIS, CLS142510, Japan). For the subcutaneous tumor model, MC38 cells (5 × 10^5^ cells) were injected into the left axillary region of mice to establish subcutaneous colorectal tumors. To evaluate whether the oral complex can induce long-term anti-tumor immune memory effect, a rechallenge tumor model was established. Firstly, MC38 cells (5 × 10^5^ cells) were subcutaneously inoculated into the left hind limb of male C57BL/6 mice to establish the primary tumor. When the tumor volume reached approximately 100 mm^3^, the mice were divided into groups G1 (PBS) and G7 (CS@CB-Lipo@5-FU/R837) for treatment according to the design, following the same treatment protocol as the *in situ* and subcutaneous tumor models. On the 15th day after treatment, all mice underwent complete surgical resection of the primary tumor and surrounding residual tissues to ensure no local recurrence. After the surgery, the same number of MC38 cells (5 × 10^5^ cells) were subcutaneously inoculated on the contralateral (right) side of the mice to simulate the tumor re-attack. After re-modeling on the right side for 15 days, record the size and weight of the tumors in these mice.

### 2.13 Biodistribution of CS@CB-Lipo@DiR

To evaluate tumor-targeting capability, orthotopic CRC mice were administered free DiR, CB-Lipo@DiR, or CS@CB-Lipo@DiR (DiR dose: 1.5 mg kg^−1^) via oral gavage. The real-time biodistribution of DiR (Ex = 748 nm, Em = 780 nm) in live mice was tracked with an IVIS imaging system under anesthesia, which was induced with 3% isoflurane and maintained at 1.5% in oxygen. At 24 h post-administration, mice were euthanized, and major organs (heart, liver, spleen, lung, and kidneys) and tumor tissues were collected for *ex-vivo* fluorescence imaging. Fluorescence intensities in the liver and tumor tissues were quantified using the IVIS Spectrum imaging software.

### 2.14 Pharmacokinetic study

Mice were orally administered free 5-FU, Lipo@5-FU/R837, CB-Lipo@5-FU/R837, or CS@CB-Lipo@5-FU/R837. Subsequently, blood samples were collected from the orbital sinus at predetermined time points (0.5 h, 1 h, 2 h, 3 h, 4 h, 8 h, 12 h, and 24 h) and centrifuged (3500 rpm, 15 min) to obtain plasma. At selected time points (2 h, 4 h, 8 h, and 24 h), mice were sacrificed and major organs were harvested, homogenized, and centrifuged (12,000 rpm, 15 min) to collect supernatants. The concentration of 5-FU in plasma and tissue samples was quantified in supernatants by HPLC.

### 2.15 *In vivo* antitumor efficacy evaluation

To assess therapeutic efficacy, both orthotopic and subcutaneous CRC models were employed. BALB/c mice bearing CT26-luc tumors or C57BL/6 mice bearing MC38 tumors were randomly divided into seven groups: PBS, 5-FU, R837, CB, Lipo@5-FU/R837, CB-Lipo@5-FU/R837, and CS@CB-Lipo@5-FU/R837. Tumor growth, body weight, and survival days were monitored throughout treatment. At the endpoint, tumors were collected for histological and immunohistochemical analyses. H&E staining was performed to observe pathological changes. TUNEL assay was used to detect apoptotic cells. Immunohistochemical staining for Ki67, CRT, and HMGB1 was conducted to evaluate tumor proliferation and ICD markers. The stained sections were analyzed and imaged using the automatic digital slide scanning system (ZEISS Axioscan 7, Germany) and CLSM.

### 2.16 *In vivo* analysis of immune cells

Tumor tissues and spleens after treatment were processed into single-cell suspensions to evaluate immune responses. the tumor tissues were minced into small pieces and digested with collagenase IV for 1 h at 37 °C, and a single-cell suspension was prepared via the mechanical trituration method. The antibodies were incubated with single-cell suspensions for flow cytometry analysis according to the manufacturer's instructions. The antibody dilutions are shown in Table S2. Flow cytometry analyses of the cells were conducted with a BD LSRFFortessa. The gating strategy for each type of immune cell is referred to Figs. S27 and S31. Following Golgi apparatus stimulation, the production of cytokines Tumor Necrosis Factor-αlpha (TNF-α) and Interferon-γamma (IFN-γ) in tumor-infiltrating cells was assessed. Additionally, tumor homogenates were analyzed by ELISA to determine the levels of TNF-α (Cat# P01580), Granzyme B (GZMB, Cat# EK1115), Interleukin-10 (IL-10, Cat# EK0417), Interleukin-6 (IL-6, Cat# EK0411), and Interleukin-12 (IL-12, Cat# EK0422) according to the manufacturer's protocol (Boster Biological Technology Co., Ltd). The same immune analysis was also conducted on the tumors in the tumor re-challenge model and the results were recorded.

To evaluate whether this oral complex could produce an immune memory effect similar to that induced by *in situ* vaccines, this study detected the tissue-resident memory T cells (TRM) -related markers in tumor tissues after treatment. After the end of treatment in the orthotopic CRC models, the mice were sacrificed and the tumor tissues were completely removed. Immunohistochemical staining with anti-CD69 and anti-CD103 antibodies was performed to label the early activation marker CD69 of memory T cells and the tissue-resident marker CD103. The number or positive area percentage of CD69^+^ cells and CD103^+^ cells were counted using ImageJ software to calculate the density of positive cells in each treatment group.

### 2.17 Biosafety assessment

Major organs (heart, liver, spleen, lung, and kidneys) were collected at the end of treatment and subjected to H&E staining to evaluate the biocompatibility of CS@CB-Lipo@5-FU/R837. The stained sections were analyzed and imaged using the automatic digital slide scanning system. In addition, blood samples were analyzed for routine hematological parameters, and serum levels of urea and creatinine were measured to assess potential systemic toxicity.

### 2.18 16S rRNA sequencing

To investigate the potential impact of CS@CB-Lipo@5-FU/R837 on gut microbiota composition, fecal samples were collected after treatment endpoint from 4 groups of mice in the orthotopic CRC-bearing mice. Genomic DNA was extracted and subjected to 16S rRNA gene sequencing (Applied Protein Technology, Shanghai, China). Sequencing libraries were analyzed to assess microbial composition, richness, and diversity among treatment groups.

### 2.19 Statistical analysis

All experiments were performed at least three times independently. Statistical analyses were conducted using GraphPad Prism 10.8 software (GraphPad Software, USA). Data are presented as mean ± standard error of the mean (SEM). Statistical significance was analyzed using the Student's t-test, one-way ANOVA, with Tukey's post hoc analysis used to determine significant differences between groups. The significance is indicated by asterisks in all figures (ns, P > 0.05; ∗*P* < 0.05; ∗∗*P* < 0.01; ∗∗∗*P* < 0.001).

## 3 Results and discussion

### 3.1 Preparation and characterization of CS@CB-Lipo@5-FU/R837

**Fig. 1:**
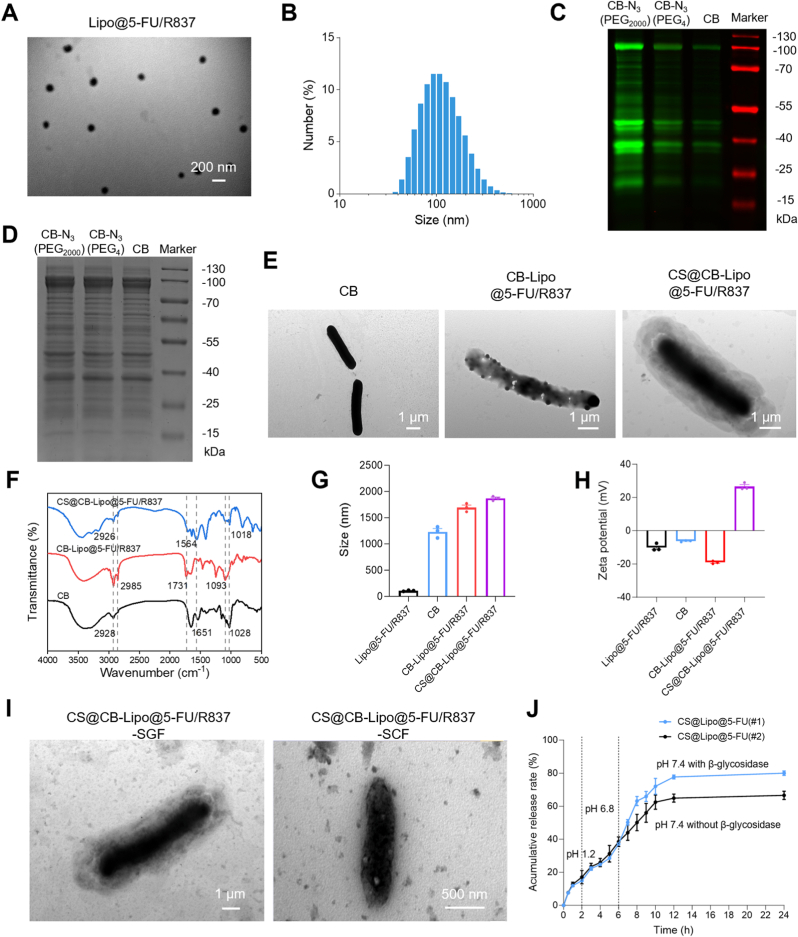
Preparation and characterization of CS@CB-Lipo@5-FU/R837. (A) TEM images of Lipo@5-FU/R837. (B) The size distribution of Lipo@5-FU/R837 determined by dynamic light scattering (DLS). (C) Fluorescent gel image of DBCO-AF488-labeled CB, CB-N_3_(PEG_2000_) and CB-N_3_ (PEG_4_). (D) Coomassie brilliant blue staining image of CB, CB-N_3_(PEG_2000_) and CB-N_3_(PEG_4_). (E) TEM images of CB, CB-Lipo@5-FU/R837 and CS@CB-Lipo@5-FU/R837. (F) FT-IR spectra of CB, CB-Lipo@5-FU/R837 and CS@CB-Lipo@5-FU/R837. (G) The size distribution of Lipo, CB, CB-Lipo@5-FU/R837 and CS@CB-Lipo@5-FU/R837. (H) Zeta potential of Lipo, CB, CB-Lipo@5-FU/R837 and CS@CB-Lipo@5-FU/R837. (I) TEM images of CS@CB-Lipo@5-FU/R837 after incubation in simulated gastric fluid (SGF) and simulated colonic fluid (SCF). (J) Cumulative release profile of 5-FU from CS@CB-Lipo@5-FU under simulated conditions with or without β-glucosidase. Data are presented as the mean ± SEM.

Nanoliposomes represent a promising platform for improving the oral delivery of combined chemotherapeutic and immunomodulatory agents [39,40]. To co-encapsulate first-line drugs for the treatment of CRC (5-FU) and TLR7 agonist R837 with good tolerance approved by FDA, DBCO-functionalized nanoliposomes (Lipo@5-FU/R837) were prepared using the thin-film hydration method. The appropriate particle size helps to improve the cellular uptake rate. The uniform particle size and appropriate particle size greatly promote the efficient endocytosis of tumor cells, which is an important basis for evaluating its use as a potential nanomedicine carrier. Characterization by transmission electron microscopy (TEM) (Fig. 1A) confirmed the formation of uniform, spherical liposomes. Dynamic light scattering measurements indicated an average hydrodynamic diameter of approximately 100 nm with a narrow polydispersity index (Fig. 1B). The drug encapsulation efficiency and loading capacity, as determined by high-performance liquid chromatography (HPLC), were calculated to be 76% (6.1%) for 5-FU, and 82% (6.58%) for R837, indicating successful and efficient co-loading of both cargoes.

It is noteworthy that strategies for labeling azide on bacterial surfaces such as chemical and metabolic labeling, are well-established and recognized for achieving excellent efficiency. For the probiotic component of the delivery system, the surface of CB (ATCC 19398) was functionalized with azide groups to enable subsequent bioorthogonal conjugation. Successful conjugation was initially suggested by ultraviolet-visible (UV-vis) spectroscopy, which exhibited characteristic wavelength shifts in the 200-300 nm range (Fig. S1). Fluorescent gel analysis with DBCO-AF488 further confirmed labeling efficiency, with N_3_-PEG_2000_-NHS linker demonstrating the superior fluorescence intensity compared to its shorter-chain counterpart (Fig. 1C–S2). Critically, coomassie blue staining revealed negligible alterations in the bacterial protein profile after azide modification, indicating preserved protein integrity and function (Fig. 1D). Based on the optimal labeling efficiency and probiotic viability, CB-N_3_ (PEG_2000_) was selected as the probiotic delivery vector for all subsequent assembly steps. The successful azide functionalization was further corroborated by confocal laser scanning microscopy (CLSM) and flow cytometry (FCM) (Fig. S3). Plate culture assays, as illustrated in Fig. S4, showed that CB-N_3_ (PEG_2000_) retained higher viability than CB-N_3_ (PEG_4_), likely due to the enhanced biocompatibility afforded by the extended PEG_2000_ spacer. The above experimental results show that the azido group is successfully modified on the surface of probiotics (CB), which lays a solid foundation for subsequent probiotic loaded nanoliposomes to target drug delivery to the lesion area of the CRC.

To enhance the loading efficiency of probiotics on drug liposomes, the DBCO groups on the surface of the liposomes and the azide groups on the upper surface of the probiotics successfully prepared a probiotic complex loaded with drug liposomes through bioorthogonal click reaction (CB-Lipo@5-FU/R837). Bioorthogonal click chemistry was employed to achieve robust conjugation between the therapeutic nanoliposomes and the probiotic vector, leveraging its high specificity and catalyst-free reaction conditions [41]. The CB-Lipo@5-FU/R837 complex was successfully formed through the reaction of CB-N_3_ (PEG_2000_) with DBCO-functionalized Lipo@5-FU/R837. The drug loading efficiencies on the bacterial surface, as quantified by HPLC, were determined to be 0.76 μg 10^−6^ CFU for 5-FU and 0.82 μg 10^−6^ CFU for R837. Notably, this bioorthogonal approach resulted in a superior drug loading efficiency compared to traditional physical adsorption methods, ensuring a higher density of liposomes anchored per probiotic carrier [42]. The resulting CB-Lipo@5-FU/R837, complex exhibited a strongly negative surface zeta potential, attributable to the combined anionic character of the liposomes and the bacterial surface. This property provided a thermodynamic driving force for subsequent coating with a cationic polymer. Chitosan (CS), a biocompatible polysaccharide, was selected to form a protective layer around the complex via electrostatic deposition [43]. Coating was performed by incubating CB-Lipo@5-FU/R837 with a CS solution under gentle stirring, leading to the formation of the final composite, CS@CB-Lipo@5-FU/R837. TEM imaging visually confirmed the encapsulation of the complex within a CS shell while showing preserved liposome attachment (Fig. 1E). Fourier-transform infrared (FT-IR) spectroscopy provided molecular evidence: the characteristic polysaccharide peak of CB (900-1300 cm^−1^) diminished following liposomes conjugation, while phospholipid double peaks emerged. Subsequent CS coating masked these phospholipid signals and introduced distinctive CS sugar-ring vibrations, with a noticeable attenuation of C-H stretching signals from the liposomal lipids (2850-2900 cm^−1^, Fig. 1F). Dynamic light scattering analysis indicated an increase in hydrodynamic diameter and a shift in zeta potential from negative to positive after CS coating (Fig. 1G–H), further confirming the formation of a cationic outer layer. Collectively, these data provide compelling evidence for the successful stepwise construction of the CS@CB-Lipo@5-FU/R837 oral platform.

For orally administered complexes, stability during gastrointestinal transit and prolonged intestinal retention are critical for effective drug delivery [44,45]. Therefore, CB-Lipo@5-FU/R837 and CS@CB-Lipo@5-FU/R837 were stored at 4 °C in an aseptic PBS solution to maintain the stability of its structure and function. The viability of CB (a), CB-Lipo@5-FU/R837 (b), and CS@CB-Lipo@5-FU/R837 (c) was assessed using plate counting and optical density measurements at 600 nm (OD_600_). A slight reduction in bacterial growth was observed after CS coating (Fig. S5), which was attributed to the intact CS shell under standard culture conditions temporarily restricting metabolic activity and replication.

To assess Lipo@5-FU/R837 and CS@CB-Lipo@5-FU/R837 potential applications in the body of the two groups of delivery system, in containing 10% FBS physical environment for 48 h serum stability test *in vitro* (Figs. S6–S7). By monitoring the dynamic changes of the hydrate particle size and zeta potential evaluation the colloid stability. Furthermore, samples were collected at the 12 h time point. TEM images showed that both Lipo@5-FU/R837 and CS@CB-Lipo@5-FU/R837 maintained good morphological stability, and no obvious aggregation or structural damage was observed. The results showed that the two groups of preparations showed good serum stability: the hydrated particle size of Lipo@5-FU/R837 was stable at 1800-1900 nm, the zeta potential was maintained at −8 to −11 mV, and there was no significant change at each time point. The particle size of CS@CB-Lipo@5-FU/R837 was stable at 1800-2100 nm, with the zeta potential remaining at +25 to +35 mV, and there was no statistically significant difference compared to the initial value at each time point. Among them, the stability of CS@CB-Lipo@5-FU/R837 is superior. Among them, CS@CB-Lipo@5-FU/R837 has better stability thanks to the protective effect of CS coating: The CS shell, as a physical barrier, can effectively inhibit the non-specific adsorption of serum proteins, prevent particle aggregation or dissociation caused by protein corona formation, thereby maintaining the dual stability of particle size and surface charge, providing a guarantee for its subsequent *in vivo* application.

The resistance of the system to the harsh gastrointestinal environment was then examined. To simulate GIT transit, CS@CB-Lipo@5-FU/R837 was sequentially incubated in simulated gastric fluid (SGF, pH = 1.2) for 2 h, and simulated colonic fluid (SCF, pH = 7.4) for 4 h. TEM images revealed that the CS coating remained largely intact in SGF, confirming effective protection. In contrast, the shell disintegrated in SCF, leading to the release of the underlying nanoparticles from CB, demonstrating the formulation's stability and enzyme-responsive release characteristics (Fig. 1I). The release behavior of liposomes in the GIT was further evaluated. CS@CB-Lipo@5-FU exhibited a sustained and site-specific release profile: less 20% of 5-FU was released in the acidic SGF and approximately 35% was released in SIF. In SCF containing β-glycosidase, the cumulative release rapidly reached 80%, significantly outperforming the control without the enzyme (Fig. 1J). These data indicate that CS@CB-Lipo@5-FU/R837 possesses strong acid resistance and colon-enzyme-triggered drug release, enabling precise colonic delivery.

Upon oral administration, the CS shell functions as a protective barrier during gastrointestinal transit. When the complex reaches the colon, the CS coating is degraded by microbial β-glucosidase, enabling site-specific release of the underlying CB-Lipo@5-FU/R837 [23,46]. The liberated probiotic vector, guided by its innate anaerobic motility and hypoxic tropism, then facilitates tumor-specific accumulation of the therapeutic payload. This targeted delivery strategy represents a significant advancement over conventional oral chemotherapy, enabling spatiotemporally controlled drug release and localized immune activation for combined chemo-immunotherapy [47].

### 3.2 Tumor cytotoxicity, cellular uptake and antitumor efficacy of CS@CB-Lipo@5-FU/R837 *in vitro*

To determine the optimal molar ratio for the synergistic induction of immune responses by 5-FU and R837, we prepared Lipo@5-FU/R837 formulations with different drug ratios for parallel evaluation. The concentration of 5-FU was fixed at 10 μM, and the molar ratios of R837 to 5-FU were set at 0.5:1, 1:1 and 2:1 respectively. Free 5-FU and free R837 were used as controls (Fig. S8). The double positive rate of CD80/CD86 on the surface of BMDCs was detected by FCM to reflect the level of DC maturation. Free 5-FU and free R837 could only induce moderate DC maturation (DC maturation rates were 9.17% and 10.5%, respectively). All combination groups were significantly superior to the single-drug groups, and the DC maturation rate induced by the 5-FU and R837 M ratio of 1:1 was the highest (21.2%), significantly higher than other ratio groups. In conclusion, when the molar ratio of 5-FU to R837 is 1:1, the best synergistic balance is achieved between chemotherapy-induced immunogenic death and the immune activation of TLR7 agonists. This ratio provides a clear optimization basis for the subsequent construction of Lipo@5-FU/R837 and CS@CB-Lipo@5-FU/R837, and also provides direct experimental evidence for the design of *in vivo* administration doses.

**Fig. 2:**
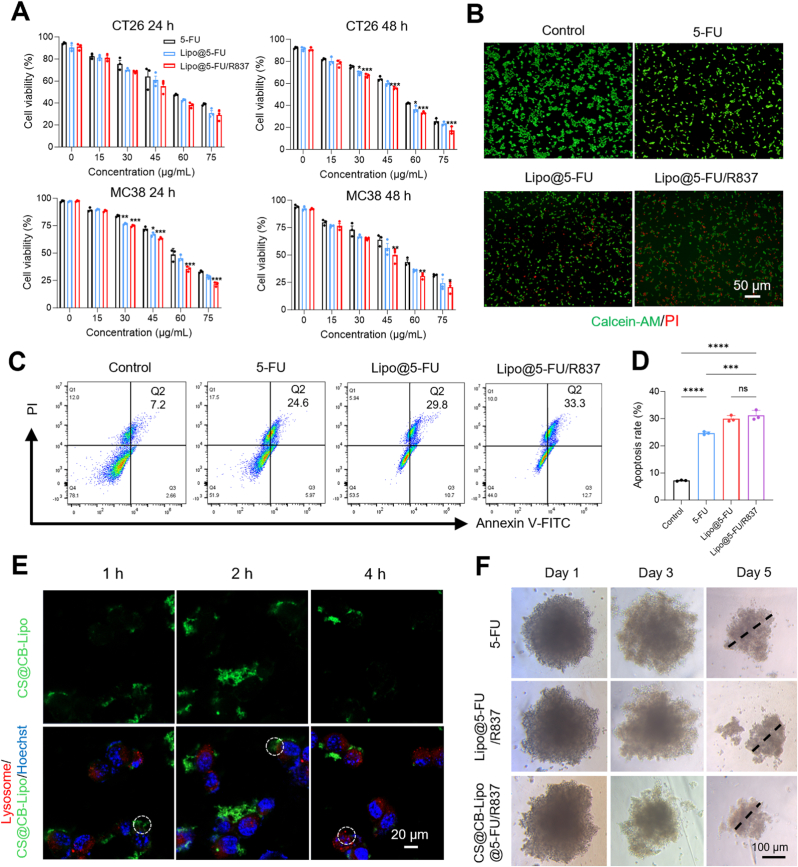
Tumor cytotoxicity, cellular uptake and antitumor efficacy of CS@CB-Lipo@5-FU/R837 *in vitro*. (A) Cell viability of CT26 cells and MC38 cells under different treatments for 24 h and 48 h using CCK-8 kit. (B) Live/dead staining of CT26 cells. Green fluorescence indicates calcein-AM staining for living cells, and red indicates propidium iodide (PI) staining for dead cells. (C) Apoptosis analysis by flow cytometry (FCM), and (D) quantitative analysis of FCM. (E) Colocalization staining of AF488-labeled CS@Lipo@AF488 (green) with lysosomes for 1, 2, and 4 h, respectively. Lysosomes were stained with Lyso-Tracker Red, and the cell nuclei were stained with Hoechst (blue). (F) The size of 3D tumor spheroids derived from CT26 cells at different time points under different treatments. Data are presented as the mean ± SEM. Statistical significance was determined using one-way ANOVA with Tukey's multiple comparisons. ns, *P* > 0.05, ∗*P* < 0.05, ∗∗*P* < 0.01, ∗∗∗*P* < 0.001, ∗∗∗∗*P* < 0.0001.

The *in vitro* antitumor efficacy of the liposomal formulation Lipo@5-FU/R837 was subsequently evaluated. As shown in Fig. 2A and Fig. S9A, Lipo@5-FU/R837 induced the most potent cytotoxic effect, whereas minimal toxicity was observed in L929 fibroblast cells, indicating a favorable cancer cell selectivity. To further visualize cell viability, CT26 and MC38 cells were stained with Calcein-AM/propidium iodide (PI) following treatment. Live/dead fluorescence imaging corroborated the cytotoxicity results, with cells treated with Lipo@5-FU/R837 displaying extensive red fluorescence, indicative of widespread cell death (Fig. 2B–S9B). The results of FCM revealed that apoptotic rate in CT26 cell reached 33.3% in the Lipo@5-FU/R837 group, higher than free 5-FU (24.6%) or R837 alone (14.6%) (Fig. 2C-D). These results indicated that Lipo@5-FU/R837, as the core therapeutic component of the delivery system, exhibited stronger antitumor activity and proapoptotic effect than a single drug.

We first examined whether CT26 cells could efficiently internalize the liposomal formulation. AF488-labeled liposomes were incubated with CT26 cells for 3 h. CLSM images (Fig. S10A) and quantitative fluorescence intensity analysis (Fig. S10B) confirmed that the liposomes were efficiently taken up by cancer cells. This was further corroborated by flow cytometry (Figs. S10C–S10D), which demonstrated a strong fluorescent signal in the treated cell population. These results confirm that the liposomal carriers can be effectively internalized by CT26 cells, setting the stage for subsequent intracellular trafficking. As endolysosomal escape is critical for ensuring that encapsulated drugs reach their cytoplasmic targets [48]. During phagocytosis of foreign substances, they are initially transported to lysosomes for digestion and processing. The intracellular trafficking of internalized liposomes was then investigated. A co-localization study was performed using Lysotracker Red for lysosomal staining and AF488-labeled liposomes (CS@Lipo@AF488). CLSM images demonstrated that at 1 h post-incubation, most of the green fluorescence (AF488) overlapped with lysosomal signals, indicating initial entrapment in endolysosomal compartments. Partial separation was observed by 2 h and by 4 h, most liposomes had escaped from lysosomes, as evidenced by a significant reduction in the co-localization coefficient (Fig. 2E and Fig. S11). This may be due to the consumption of H^+^ in the lysosome of CS, accelerating the entry of cytosolic hydrogen into the lysosome, increasing the osmotic pressure in the lysosome, and finally leading to the rupture of the lysosomal membrane and the release of CS@Lipo@AF488. The above experimental results indicate that after the drug-loaded liposomes are engulfed by cancer cells, they first enter the lysosomes, and then can escape from the lysosomes into the cytoplasm, thereby exerting their therapeutic effect.

The deep tissue penetration capacity was further validated using three-dimensional (3D) tumor spheroids as a physiologically relevant model. The inhibitory effects of free 5-FU, Lipo@5-FU/R837, and CS@Lipo@5-FU/R837 on tumor spheroids were evaluated over 5 days. Bright-field microscopy monitoring revealed that while all treatment groups induced varying degrees of spheroid shrinkage, the CS@Lipo@5-FU/R837 elicited the most sustained and pronounced volume reduction (Fig. 2F). This superior efficacy suggested that CS functionalization significantly improved the tumor-penetrating capability.

This finding was further supported using CT26-luciferase (CT26-luc) spheroids, where treatment-induced cytotoxicity was quantified by bioluminescence intensity measurement via the In Vivo Imaging System (IVIS). Consistent with the morphological observations, CS@Lipo@5-FU/R837 treatment resulted in continuous shrinkage and luminescence decline, confirming enhanced tumor penetration and efficacy (Fig. S12). The experimental results show that CS@CB-Lipo@5-FU/R837 has strong toxicity of tumor cells, and achieves lysosomal escape after being taken up by cells, thus effectively inhibiting the formation and growth of tumors.

### 3.3 *In vitro* immune activation and ICD induction by CS@CB-Lipo@5-FU/R837

**Fig. 3:**
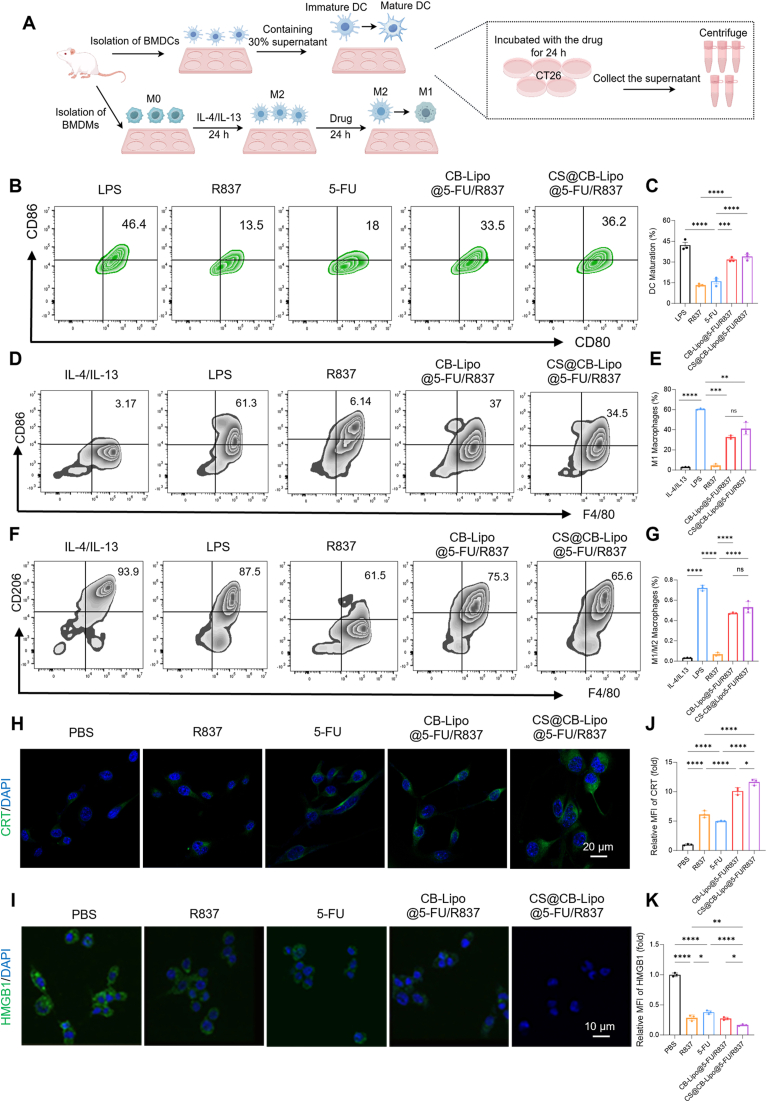
*In vitro* immune activation and ICD induction by CS@CB-Lipo@5-FU/R837. (A) Schematic illustration of BMDCs activation and BMDMs repolarization assays. (B) Representative flow cytometry analysis of BMDCs activation, and (C) quantitative analysis of matured BMDCs. (D) Representative flow cytometry analysis of M1 markers (CD86) in BMDMs, and (E) quantitative analysis of M1 macrophages proportion. (F) Representative flow cytometry analysis of M2 markers (CD206) in BMDMs, and (G) quantitative analysis of M1/M2 ratios. (H) CLSM images of exposure, Immunofluorescence staining images of CRT (green) and (I) HMGB1 in CT26 cells under different treatments. The cell nuclei were stained with DAPI (blue). (J) Statistical analysis of the relative mean fluorescence intensity (MFI) of CRT and (K) HMGB1. Data are presented as the mean ± SEM. Statistical significance was determined using one-way ANOVA with Tukey's multiple comparisons. ns, *P* > 0.05, ∗*P* < 0.05, ∗∗*P* < 0.01, ∗∗∗*P* < 0.001, ∗∗∗∗*P* < 0.0001.

The TME, characterized by immature dendritic cells (DCs) and M2-polarized macrophages, represents a major barrier in CRC therapy [49,50]. Reversing this immunosuppression is critical for effective treatment. The capacity of CS@CB-Lipo@5-FU/R837 to stimulate DC maturation and macrophage repolarization-key steps in initiating anti-tumor immunity was therefore evaluated. As outlined in Fig. 3A, bone marrow-derived DCs (BMDCs) and macrophages (BMDMs) were exposed with different formulations. FCM analysis showed that BMDCs treated with the supernatant from the CS@CB-Lipo@5-FU/R837 group exhibited a significantly higher percentage of CD80^+^CD86^+^ cells among CD11c^+^ populations, compared to those treated with free drugs (Fig. 3B–C). This result demonstrated that the complex effectively promoted DC maturation, enhancing their potential for T cell activation.

The ability of CS@CB-Lipo@5-FU/R837 to repolarize macrophages from the immunosuppressive M2 phenotype to the pro-inflammatory M1 state-a key step in reversing tumor immunosuppression was also examined [51,52]. Following M2 polarization induced by IL-4 and IL-13, BMDMs treated with CS@CB-Lipo@5-FU/R837 showed significant upregulation of the M1 marker CD86 and downregulation of the M2 marker CD206 on F4/80^+^ cells (Fig. 3D–F). This phenotypic shift resulted in a substantially increased M1/M2 ratios (Fig. 3G), indicating potent repolarization activity. Cytokine profiling of culture supernatants by ELISA further supported these findings, demonstrating elevated levels of TNF-α, IL-6, and IL-12, along with reduced secretion of IL-10 (Fig. S13). These findings demonstrated that the complex not only promoted DC maturation but also reprograms macrophage populations, collectively establishing a pro-inflammatory milieu conducive to antitumor immunity.

Beyond immune cell modulation, the induction of ICD by 5-FU released from the delivery system was investigated, as ICD converts dying tumor cells into endogenous antigen sources. Treatment with CS@CB-Lipo@5-FU/R837 triggered all ICD hallmarks: surface exposure of calreticulin (CRT), extracellular release of high mobility group box 1 protein (HMGB1), and ATP efflux. CLSM analysis revealed significantly increased CRT fluorescence on the cell membrane and decreased HMGB1 signal in the cytoplasm following treatment with the complex compared to free drug groups (Fig. 3H–K), confirming efficient exposure of damage-associated molecular patterns (DAMPs). Moreover, ATP quantification in the supernatant showed substantially increase in extracellular ATP (Fig. S14), further validating ICD occurrence among these. The Western blot results further confirmed the observations made by confocal microscopy: Consistent with the confocal observations showing enhanced CRT membrane exposure and increased HMGB1 release, quantitative WB analysis indicated that the intracellular CRT expression was upregulated, HMGB1 retention was reduced, and the changes were most significant in the CS@CB-Lipo@5-FU/R837 group (Figs. S15 and S16). The two methods mutually corroborated, providing a dual confirmation of the efficient ICD induction ability of this delivery system through protein localization and expression levels. The spatiotemporally coordinated release of tumor antigens and DAMPs, together with DC activation and macrophage repolarization, established a favorable microenvironment for *in situ* vaccination, effectively linking innate and adaptive antitumor immunity.

### 3.4 *In vivo* biodistribution and pharmacokinetics of CS@CB-Lipo@5-FU/R837

**Fig. 4:**
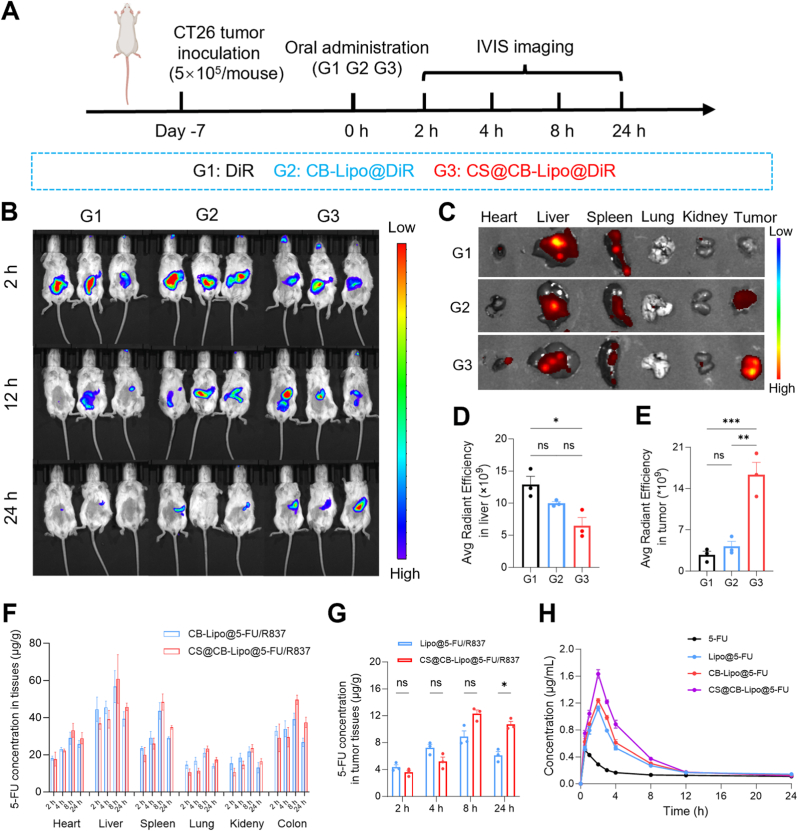
*In vivo* biodistribution and pharmacokinetics of CS@CB-Lipo@5-FU/R837. (A) Schematic diagram for verifying *in vivo* distribution of orthotopic CRC model. (B) *In vivo* bioluminescence images of tumors in CT26-luc tumor-bearing mice at 2, 12, and 24 h after after orally administrated with free DiR, CB-Lipo@ DiR and CS@CB-Lipo@ DiR. (C) *Ex vivo* fluorescence images of major organs and tumors from CT26 tumor-bearing mice at 24 h after orally administrated. (D) Quantitative analysis of fluorescence intensity in the liver and (E) tumor tissues (n = 3). (F) The content of 5-FU distribution in different organs and (G) tumor tissues. (H) Plasma concentration-time profile of 5-FU (n = 3). Data are presented as the mean ± SEM. Statistical significance was determined using one-way ANOVA with Tukey's multiple comparisons. ns, *P* > 0.05, ∗*P* < 0.05, ∗∗*P* < 0.01, ∗∗∗*P* < 0.001, ∗∗∗∗*P* < 0.0001.

To evaluate the *in vivo* fate and targeting efficiency of the delivery system, an orthotopic CT26-luc colorectal cancer model was established. Mice were orally administered free DiR, CB-Lipo@DiR, or CS@CB-Lipo@DiR (DiR dose: 1.5 mg kg^−1^), and real-time fluorescence imaging was performed at 2 h, 4 h, 8 h, and 24 h post-administration with the IVIS (Fig. 4A). In the free DiR group, fluorescence intensity in the abdominal region peaked at 2 h and declined rapidly thereafter, with minimal signal detected at 24 h (Fig. 4B). In contrast, both CB-Lipo@DiR and CS@CB-Lipo@DiR groups showed sustained abdominal fluorescence through 24 h. *Ex vivo* imaging of major organs and tumors collected at 24 h demonstrated that CS@CB-Lipo@DiR achieved the highest tumor-specific accumulation a with reduced off-target distribution in non-target organs such as the liver (Fig. 4C–E). As shown in Fig. S17A, further analysis of intestinal fluorescence distribution revealed that in the free DiR group, the fluorescent signal was rapidly cleared from the gastrointestinal tract, with only weak residual signals in the stomach and the end of the colon, failing to achieve long-term retention. In the CB-Lipo@DiR group, the fluorescent signal was scattered in the small intestine and parts of the colon, and the intensity decayed rapidly over time. In contrast, the CS@CB-Lipo@DiR group exhibited excellent intestinal retention performance, with fluorescence highly enriched in the terminal ileum, the entire colon, and notably accumulated in the cecum. The mucoadhesive property of CS effectively prolonged the residence time of the formulation in the lower digestive tract, creating a prerequisite for targeted enrichment of the carrier to colorectal tumor lesions. To quantitatively verify the tumor-targeting enrichment capability of the carrier, we further performed tissue colonization quantification using the colony plating method (Figs. S17B–S17C). During the *in vitro* pre-treatment stage, the bacterial suspensions were uniformly prepared to ensure that free CB and CS@CB-Lipo@5-FU/R837 contained equal amounts of viable bacteria, thereby eliminating the interference of initial bacterial count differences. After oral administration of the same viable bacterial dose to all groups, normal colonic mucosa and colorectal tumor tissues were collected, homogenized, serially diluted, and plated on selective agar plates. The colonization levels of bacteria in different tissues were quantified by colony counting. The quantitative results were consistent with the *in vivo* fluorescence imaging observations: CS@CB-Lipo@5-FU/R837 exhibited significantly higher bacterial colonization in tumor tissues than in paired normal colonic mucosa, whereas the non-CS-coated carrier showed no obvious difference in accumulation between tumor and normal intestinal wall. These quantitative data directly demonstrate that the prolonged intestinal retention conferred by CS modification, together with the inherent tumor-colonizing ability of CB, endows CS@CB-Lipo@5-FU/R837 with highly efficient active targeting and accumulation properties in CRC. Tissues distribution analysis further confirmed that CS@CB-Lipo@5-FU/R837 resulted in significantly higher 5-FU accumulation in colon and tumor tissues compared to the non-CS-coated control at 2, 4, 8, and 24 h post-administration (Fig. 4F–G). This suggested that, following oral administration, part of the system entered systemic circulation while the majority was specifically retained in the colon via CS infiltration and CB-mediated targeting. Pharmacokinetic analysis of plasma samples collected at 0.5 h, 1 h, 2 h, 3 h, 4 h, 8 h, 12, and 24 h showed that all liposomal formulations prolonged the time to reach maximum plasma concentration (*T*_max_) compared to free 5-FU (50 mg kg^−1^). Notably, CS@CB-Lipo@5-FU/R837 exhibited the highest peak concentration (*C*_max_) and the largest area under the curve (*AUC*) (Fig. 4H–Table S1), indicating enhanced systemic exposure and stability. The targeted colon accumulation was directly linked to the functional presence of our probiotic vector. Homogenization of colon tumor tissue followed by plate culture demonstrated that the CS@CB-Lipo@5-FU/R837 group yielded the highest number of bacterial colonies (Fig. S18), confirming the successful retention of viable CB within the tumor niche. Together, these results demonstrated that the CS coating endowed the system with enhanced gastrointestinal stability and prolonged circulation, while the enzyme-responsive CS degradation in in the colon and subsequent CB-mediated targeting work synergistically to achieve spatiotemporally controlled, tumor-specific drug delivery.

### 3.5 Anticancer efficacy and safety profile of CS@CB-Lipo@5-FU/R837 in mice with orthotopic CRC and subcutaneous CRC

**Fig. 5:**
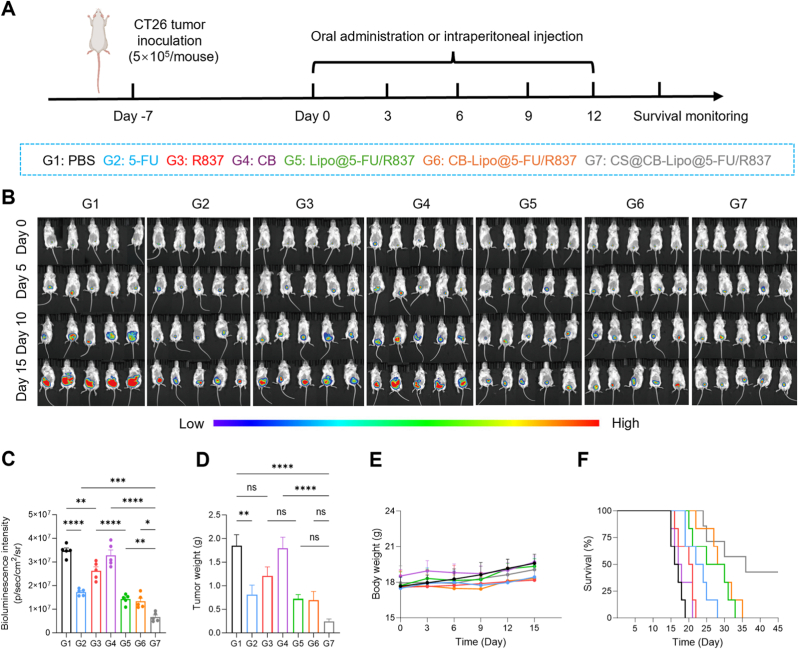
*In vivo* anti-tumor efficacy of CS@CB-Lipo@5-FU/R837 in CT26 tumor-bearing mice. (A) Schematic illustrating the establishment of the orthotopic CRC model and the treatment timeline. (B) *In vivo* bioluminescence images acquired every 5 days and (C) quantitative photon flux measurement at the end point. (D) Tumor weight measured after dissection. (E) Survival curves and (F) body weight changes of orthotopic CRC mice in different treatment groups (n = 5). Data are presented as the mean ± SEM. Statistical significance was determined using one-way ANOVA with Tukey's multiple comparisons. ns, *P* > 0.05, ∗*P* < 0.05, ∗∗*P* < 0.01, ∗∗∗*P* < 0.001, ∗∗∗∗*P* < 0.0001.

Building upon the favorable *in vitro* and biodistribution profiles, the *in vivo* therapeutic potential of CS@CB-Lipo@5-FU/R837 was further evaluated in an orthotopic CT26-luc CRC model. Mice were randomly divided into seven groups (G1-G7) following the scheme in Fig. 5A, receiving PBS, free 5-FU, free R837, CB alone, Lipo@5-FU/R837, CB-Lipo@5-FU/R837, or CS@CB-Lipo@5-FU/R837. Treatments were initiated 7 days after tumor inoculation and administered orally every two days for five doses, except for G2 and G3, which received intraperitoneal injections. *In vivo* bioluminescence imaging (Fig. 5B) revealed that CS@CB-Lipo@5-FU/R837 (G7) elicited the strongest suppression of tumor growth (Fig. 5C–D). Body weight remained stable across all groups during the treatment period (Fig. 5E). Prolonging the survival period of patients is the ultimate goal of cancer treatment. The survival rate results show that a large proportion of the mice treated with G1, G3 and G4 groups died. During the treatment stage, the median survival time of the patients was only 17 days. However, the survival period of the G7 group was significantly prolonged, with a median survival time of more than 30 days (Fig. 5F). Upon study termination, *ex vivo* analysis confirmed that tumors and spleens from the G7 group were the smallest and lightest among all groups (Figs. S19–S20). Notably, CB alone (G4) showed no significant antitumor effect, underscoring that therapeutic efficacy depended on the integrated delivery system rather than the bacterial vector itself. Histopathological analysis provided further mechanistic insights. Hematoxylin and eosin (H&E) staining confirmed extensive necrosis within tumors of the G7 group (Fig. S20). Immunofluorescence staining showed decreased Ki67 expression and increased TUNEL-positive cells in the same group, indicating suppressed proliferation and enhanced apoptosis. The highly concentrated chemotherapeutic agent (5-FU) induces ICD, effectively transforming the tumor into an “endogenous antigen reservoir' and releasing tumor-associated antigens (TAAs), thereby establishing a robust foundation for effective immune activation [53]. Concurrently, R837 is released within TME, facilitating the maturation of DCs and the polarization of macrophages, thereby enhancing the immune response. Immunohistochemistry for ICD marker (CRT and HMGB1) showed significantly increased expression in G7 group. indicating potent ICD activation (Fig. S21).

**Fig. 6:**
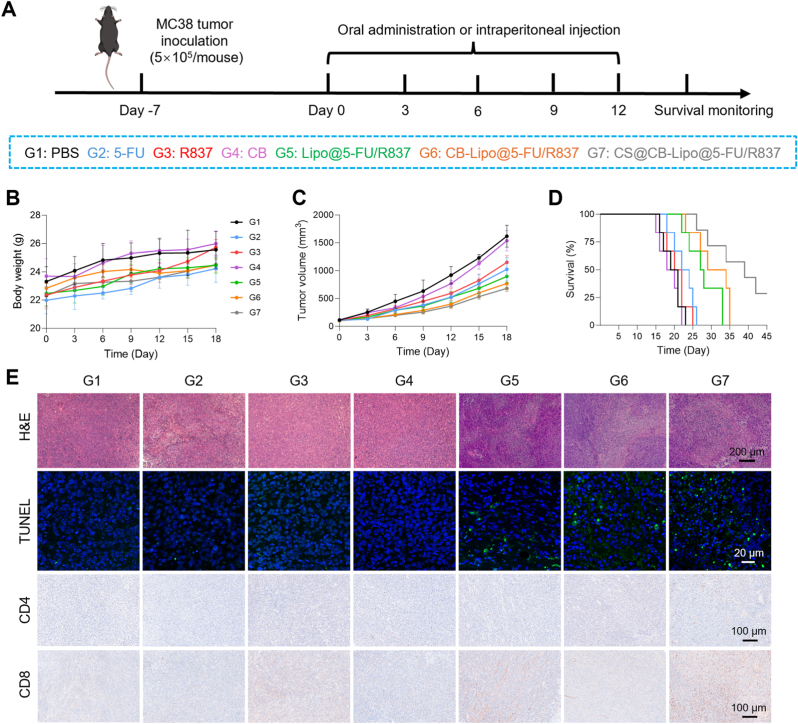
*In vivo* anti-tumor efficacy of CS@CB-Lipo@5-FU/R837 in MC38 tumor-bearing mice. (A) Schematic illustrating the establishment of the subcutaneous CRC mice and the treatment timeline. (B) Body weight changes, (C) tumor volume, and (D) survival curves of subcutaneous CRC mice in different treatment groups (n = 6). (E) Representative H&E staining, TUNEL staining, CD4 and CD8 immunohistochemistry images in different treatment groups of tumor tissues.

Following the demonstration of therapeutic efficacy in the orthotopic CRC model, the antitumor performance of CS@CB-Lipo@5-FU/R837 was further evaluated in a subcutaneous MC38 tumor model using C57BL/6 mice. The treatment protocol mirrored that of orthotopic CRC model, as depicted in Fig. 6A. Mice treated with CS@CB-Lipo@5-FU/R837 (G7) exhibited the most effective suppression of tumor growth and a significant survival advantage, with no appreciable change in body weight across all groups (Fig. 6B–D, S22). H&E staining revealed that CS@CB-Lipo@5-FU/R837 significantly induced tumor regression in the subcutaneous model (Fig. 6E). TUNEL staining further indicated pronounced apoptosis in G7 tumors, while minimal apoptotic signals were detected in G1 (PBS) and G4 (CB alone) groups, consistent with the H&E findings. To characterize the immune response activated by the treatment, tumor-infiltrating CD4^+^ and CD8^+^ T cells were analyzed by immunohistochemistry (Fig. 6E). Quantitative analysis of the IHC sections was performed in Fig. S23. The highest levels of CD4^+^ and CD8^+^ T cell infiltration was observed in the G7 group, with moderate activation also detected in groups G5 and G6. These results indicated that the complex not only directly induced tumor cell death but also stimulated an adaptive immune response, contributing to sustained tumor control. The treatment also demonstrated a favorable safety profile in this model.

Good biocompatibility is of vital importance for the new oral drug delivery system. Histopathological examination (H&E staining) in mice with orthotopic CRC or subcutaneous CRC of major organs showed no significant abnormalities, indicating the system's capacity to inhibit metastatic progression (Figs. S24–S25). Hematological (White blood cell, Red blood cell, Platelet) and biochemical parameters, including alanine aminotransferase (ALT), aspartate aminotransferase (AST), blood urea nitrogen (BUN) and creatinine (CREA), were within normal ranges across all groups (Fig. S26). Overall, this innovative oral delivery system consolidates an oral *in situ* vaccine into a unified, continuous platform, presenting a promising strategy for the treatment of both primary and subcutaneous CRC. In summary, these results suggested that the oral composite was effective across different CRC models and offered insights for the treatment of various models.

### 3.6 CS@CB-Lipo@5-FU/R837 reshapes immunosuppressive microenvironment and potentiates antitumor immunity

**Fig. 7:**
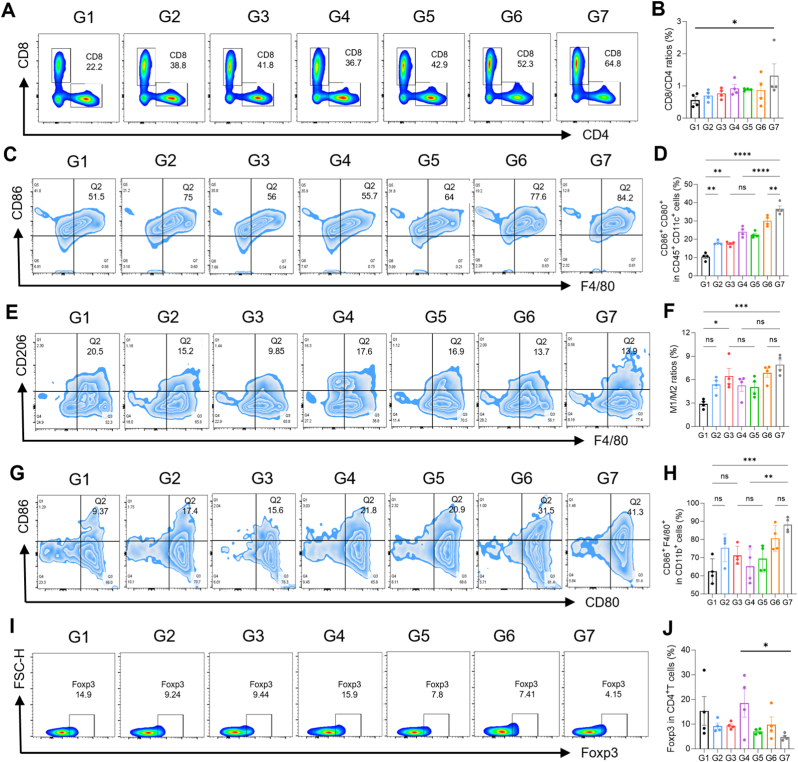
CS@CB-Lipo@5-FU/R837 reshapes immunosuppressive microenvironment and potentiates antitumor immunity. (A) Representative flow cytometry analysis of CD8^+^ T cells in tumor tissues, and (B) quantitative analysis of CD8^+^/CD4^+^ ratios. (C) Representative flow cytometry analysis of CD206 and (D) CD86 in F4/80^+^ in tumor tissues. (E) Quantitative analysis of CD86^+^ cells and (F) M1/M2 ratios. (G) Representative flow cytometry analysis of DCs maturation, and (H) quantitative analysis of matured DCs in spleen. (I) Percentage of Foxp3^+^ cells among CD4^+^ T cells, and (J) quantitative analysis of Foxp3^+^ cells among CD4^+^ T cells in spleen. Data are presented as the mean ± SEM. Statistical significance was determined using one-way ANOVA with Tukey's multiple comparisons. ns, *P* > 0.05, ∗*P* < 0.05, ∗∗*P* < 0.01, ∗∗∗*P* < 0.001, ∗∗∗∗*P* < 0.0001.

The oral delivery platform was designed to achieve spatiotemporally coordinated delivery of an ICD inducer (5-FU) and an immune adjuvant (R837). It was hypothesized that this spatiotemporally coordinated “antigen release-immune activation' mechanism would effectively simulate local *in situ* vaccination, augmenting CD8^+^ T cell-mediated cytotoxicity and tumor infiltration [3,54], while initiating a robust systemic antitumor immune response [27,55]. To test this hypothesis, immune activation was assessed in the orthotopic CRC model following treatment with CS@CB-Lipo@5-FU/R837. Analysis of tumor-infiltrating lymphocytes showed that the proportion of CD8^+^ cytotoxic T cells increased from 22% to 55%, accompanied by an elevated CD8/CD4 ratios (Fig. 7A and B, S28), indicating a shift toward a cytotoxic immune phenotype. An expanded population of CD44^+^CD62L^−^ CD8^+^ T cells was also observed (Fig. S29), suggesting the formation of effector memory T cells capable of mediating rapid antitumor responses.

Tumor-associated macrophages (TAMs) were further examined to evaluate microenvironmental reprogramming [56].In the CS@CB-Lipo@5-FU/R837 group (G7), M2-type TAMs (CD206^+^) decreased significantly, while M1-type TAMs (CD86^+^) increased from 51.5% to 84.2%, resulting in the highest M1/M2 ratio among all groups (Fig. 7C–F, S30). These changes reflected effective repolarization of TAMs from a pro-tumorigenic to an antitumor phenotype, contributing to the reversal of immunosuppression in the TME. Systemic immune activation was also evaluated in the spleen. FCM analysis showed an increase proportion of activated dendritic cells (CD80^+^CD86^+^) (Fig. 7G–H) and a concurrent reduction in regulatory T cells (Tregs, Foxp3^+^CD4^+^) in spleen, leading to an elevated CD8^+^ T/Tregs ratios. Meanwhile, the same trend was also observed in tumors: dendritic cells (CD80^+^CD86^+^) significantly increased and regulatory T cells (Tregs, Foxp3^+^CD4^+^) markedly decreased (Figs. S32 and S33). Moreover, the proportion of activated CD8^+^ T cells in the spleen and the CD8/CD4 ratios also remarkably increased (Fig. S34), indicating dual immune modulation: enhancing cytotoxic cells and suppressing immunosuppressive populations. Although enhanced DC maturation and reduced Treg infiltration were confirmed within tumor tissues, immune profiling of tumor-draining lymph nodes was not performed in the current study. Considering the central role of TDLNs in antigen presentation and T-cell priming, future studies will include systematic analyses of DC activation, Treg modulation, and memory T-cell generation within TDLNs to further elucidate the mechanisms underlying the *in situ* vaccine effect of CS@CB-Lipo@5-FU/R837.

Additionally, cytotoxic T lymphocytes (CTLs) can secrete IFN-γ and TNF-α, enhancing MHC-I-mediated antigen presentation, suppressing tumor proliferation, and recruiting other immune cells [57]. FCM analysis of tumor tissues revealed markedly elevated levels of TNF-α^+^ and IFN-γ^+^ CD8^+^ T cells (Figs. S35A–S35B). ELISA further confirmed a 3.21-fold increase in granzyme B (GZMB) secretion in the G7 group compared to the PBS (G1) group (Fig. S35C), indicating enhanced cytotoxic T cell activity. Furthermore, levels of pro-inflammatory cytokines TNF-α, IL-6 and IL-12 were elevated, while the anti-inflammatory cytokine IL-10 was reduced (Figs. S35D–S35G), consistent with the observed immune activation profile.

To verify the long-term anti-tumor immune memory induced by CS@CB-Lipo@5-FU/R837, this study detected the expression levels of immune memory-related markers CD69 and CD103 in tumor tissues by IHC staining. CD69 is a key molecule for early T-cell activation and memory formation, while CD103 is a characteristic marker of tissue-resident memory T cells (TRM). The combined detection of the two can effectively evaluate the establishment of persistent immune surveillance in the tumor microenvironment, which is the core basis for inhibiting tumor recurrence and distant metastasis. IHC staining showed (Figs. S36 and S37): In the G7 (CS@CB-Lipo@5-FU/R837) group, there was extensive brown positive staining in tumor tissues, and positive cells were densely distributed in the tumor parenchyma and stroma regions; while in the G1 (PBS) group, almost no positive signals were observed. Quantitative analysis further indicated that compared with the G1 control group, the proportions of CD69 and CD103 positive areas in tumor tissues of all treatment groups were significantly increased. Among them, the expression levels of CD69 and CD103 in the G7 group reached the peak, with positive rates of approximately 69.8% and 65.42%, respectively, significantly higher than those of the G5 and G6 groups. These results indicate that this formulation can effectively induce the infiltration of CD69^+^ activated T cells and CD103^+^ TRM cells in tumor tissues, successfully establishing a long-term immune memory microenvironment. CD103^+^ TRM cells have the characteristic of tissue residence and can survive long-term in the tumor site, and they can quickly and strongly respond to re-challenge with tumor antigens. They are the key effector population for inhibiting tumor recurrence and distant metastasis. The high expression of CD69 suggests that T cells are in a continuously activated state, enhancing their ability to recognize and kill tumor cells. Therefore, CS@CB-Lipo@5-FU/R837 induces the infiltration of CD69^+^/CD103^+^ immune memory cells, establishing a tumor-specific immune memory. This finding provides direct immunological evidence for explaining the significant inhibitory effect observed in the distant metastasis model, further supporting its systemic anti-tumor effect through long-term immune memory.

To further verify the long-term anti-tumor immune memory and distant metastasis inhibition effect induced by CS@CB-Lipo@5-FU/R837, a tumor rechallenge model was established, and FCM was used to perform multi-dimensional quantitative assessment of the immune responses in the rechallenged tumor tissues. After the primary subcutaneous tumor was surgically resected and treatment was completed, MC38 cells were re-inoculated subcutaneously on the contralateral side of the mice to simulate tumor recurrence. When the rechallenged tumors reached an appropriate size, the tumor tissues were isolated and prepared into single-cell suspensions for FCM analysis. The results showed that the growth of rechallenged tumors in the CS@CB-Lipo@5-FU/R837 group (G7) was significantly inhibited, which was highly consistent with the CD69/CD103 immunohistochemistry data, further confirming that this formulation can establish tumor-specific immune memory (Fig. S38). FCM analysis first evaluated the infiltration level of effector T cells. Compared with the control group, the proportion of CD8^+^ T cells (CD3^+^CD8^+^) in the rechallenged tumors of the G7 group was significantly increased, and the CD8^+^/CD4^+^ T cell ratio was also markedly elevated, indicating the predominant accumulation of CTLs in the tumor site (Fig. S39). Subsequently, the status of antigen-presenting cells and innate immune cells was analyzed: the proportion of mature DCs (CD80^+^CD86^+^) was significantly increased in the G7 group (Fig. S40); meanwhile, macrophages were repolarized toward the M1 phenotype, as evidenced by an increased proportion of CD86^+^ M1-type macrophages and a decreased proportion of CD206^+^ M2-type macrophages (Fig. S41). In addition, the proportion of regulatory T cells (Foxp3^+^CD4^+^) was significantly reduced in the G7 group (Fig. S42). Functional molecule detection showed that the expression level of GZMB in CD8^+^ T cells reached a peak (Fig. S43), and the secretion levels of IFN-γ and TNF-α in the rechallenged tumor tissues were also significantly higher than those in other treatment groups (Figs. S44–S45). These results demonstrate that CS@CB-Lipo@5-FU/R837 not only effectively reverses the immunosuppressive microenvironment and promotes DC maturation and macrophage repolarization toward the M1 phenotype, but also enhances the infiltration and effector functions of CD8^+^ T cells, thereby confirming, at the immune cell level, the long-term anti-tumor immune memory and distant metastasis inhibition capacity induced by this formulation. Previous studies have demonstrated that orally administered anaerobic probiotics can translocate from the intestinal tract and preferentially colonize hypoxic tumor tissues. Therefore, the antitumor efficacy observed in the subcutaneous CRC model may be attributed to the intrinsic tumor-homing capability of CB following oral administration. Future studies will focus on directly tracking bacterial biodistribution in distant tumors to further elucidate this mechanism.

In summary, CS@CB-Lipo@5-FU/R837 effectively remodeled tumor immune microenvironment (TIME) by increasing CD8^+^ T cell infiltration and cytotoxicity, promoting M1 macrophage polarization, activating dendritic cells, and reducing immunosuppressive Tregs. This comprehensive immunomodulation creates a “vaccine-like' microenvironment *in situ*, thereby triggering robust systemic and local antitumor immune responses and providing a novel delivery strategy for oral chemo-immunotherapy. More importantly, the agent successfully induces tumor-specific immune memory, as evidenced by a significant increase in CD69^+^CD103^+^ TRM within the tumor tissue, and exhibits strong inhibition of secondary tumor challenge in a rechallenge model. These findings demonstrate that CS@CB-Lipo@5-FU/R837not only effectively eliminates primary tumors but also establishes long-term immune memory, thereby suppressing tumor recurrence and distant metastasis. Collectively, this oral delivery system offers a highly promising strategy for oral chemoimmunotherapy by synergizing chemotherapy, immune activation, and immune memory induction.

### 3.7 Regulation of gut microbiota by CS@CB-Lipo@5-FU/R837

The intestinal microenvironment and its dynamic crosstalk with the TME play pivotal roles in the initiation, progression, and metastasis of CRC [58]. As a common probiotic, CB has been reported to modulate gut ecosystem through the production of short-chain fatty acids (SCFAs) and by altering the balance between commensal and pathogenic bacteria, processes linked to the regulation of local and systemic immunity [28]. Although CB was primarily employed as a biological targeting vehicle in this study, its inherent probiotic properties prompted investigation into whether the CB-based complex could alter the composition and abundance of gut microbiota in CRC-bearing mice [30,59,60].

**Fig. 8:**
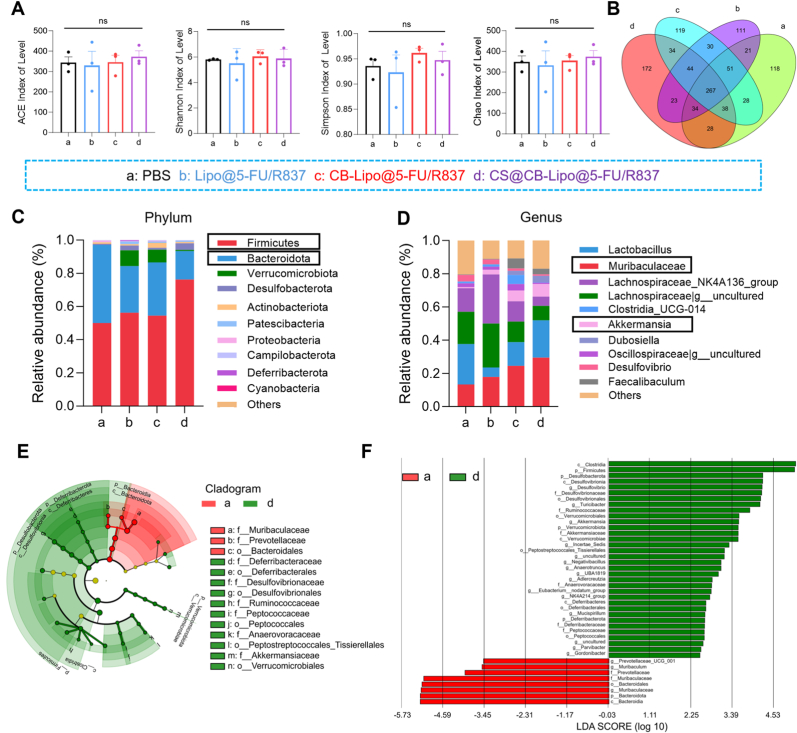
CS@CB-Lipo@5-FU/R837 modulates gut microbiota composition. (A) Microbial α-diversity showing microbial community richness and diversity based on ACE, Shannon, Simpson, and Chao indices. (B) Venn diagram displaying the number of shared and unique OTUs among four groups. (C) Relative abundance profiles of gut microbiota at the phylum, and (D) genus levels across four groups. (E) LEfSe taxonomic cladogram illustrating taxa with significant differential abundance among four groups. (F) LDA score histogram identifying taxa with an LDA score >3.0, indicating significantly higher relative abundance in the corresponding group compared with others (n = 3). Data are presented as the mean ± SEM. Statistical significance was determined using one-way ANOVA with Tukey's multiple comparisons. ns, *P* > 0.05.

Fecal samples were collected from orthotopic CRC mice after treatment with PBS, Lipo@5-FU/R837, CB-Lipo@5-FU/R837, and CS@CB-Lipo@5-FU/R837), and analyzed via 16S rRNA gene sequencing. Analysis of α-diversity indices (ACE, Shannon, Simpson, and Chao) showed no significant differences among the four groups (Fig. 8A), indicating that the treatments did not substantially affect overall microbial richness or diversity. However, Venn diagram of operational taxonomic units (OTUs) revealed that the CS@CB-Lipo@5-FU/R837 group harbored a greater number of unique species compared to the PBS control (Fig. 8B), suggesting qualitative enrichment of the microbial community. Further analysis at the phylum level revealed a significant shift in microbial composition. The relative abundance of *Firmicutes* decreased markedly, while *Bacteroidetes* increased, resulting in a lower *Firmicutes/Bacteroidetes* (F/B) ratios (Fig. 8C–S46A-S46B). This shift toward a reduced F/B ratios is generally indicative of a more favorable gut microbial profile. Compared with the PBS group, the CS@CB-Lipo@5-FU/R837 treatment group exhibited a significant reduction in the F/B ratios, along with enrichment of beneficial genera such as *Lactobacillus* and *Akkermansia* at the genus level (Fig. 8D). This compositional shift in the gut microbiota is closely associated with increased production of SCFAs, particularly acetate, propionate, and butyrate. As key metabolites of the gut microbiota, SCFAs can activate G protein-coupled receptors (GPR41 and GPR43) [61] and inhibit histone deacetylases (HDACs), thereby regulating intestinal epithelial barrier function, anti-inflammatory responses, and antitumor immunity [62,63]. Therefore, the “probiotic-shift” of gut microbiota composition induced by CS@CB-Lipo@5-FU/R837 likely exerts synergistic anti-tumor effects in CRC therapy through the aforementioned metabolism-dependent immunomodulatory mechanisms. This interpretation establishes a functional causal link between microbiota changes and therapeutic efficacy.

Structural differences in microbial communities were further assessed by β-diversity analysis. Principal coordinate analysis (PCoA) showed clear separation of the CS@CB-Lipo@5-FU/R837 group from other groups along PCoA1 and PCoA2 axes (Fig. S46C), indicating distinct community structure. This finding was supported by Linear Discriminant Analysis Effect Size (LEfSe), which identified specific bacterial taxa from phylum to species level that were significantly enriched in the CS@CB-Lipo@5-FU/R837 group (Fig. 8E–F).

Collectively, these results suggested that although the overall microbial diversity was maintained, CS@CB-Lipo@5-FU/R837 treatment induced a compositional shift toward a more favorable gut microbial profile, which may contribute to its therapeutic efficacy in CRC.

## 4 Conclusions

In this study, an administered composite (CS@CB-Lipo@5-FU/R837) was developed to overcome the challenges of low targeting efficiency and inadequate tumor accumulation in CRC therapy. The system integrated CB as a biotrophic vector, leveraging its strict anaerobicity and innate tumor-homing capability. Nanoliposomes co-loaded with 5-fluorouracil (5-FU) and the TLR7 agonist R837 were site-specifically conjugated to CB via bioorthogonal chemistry, and encapsulated within CS coating to provide gastrointestinal protection and enable enzyme-responsive release in the colon.

Upon colonic arrival, the CS shell was degraded by β-glucosidase, leading to the release of the CB-guided therapeutic cargo. This facilitated tumor-specific drug delivery, where localized high concentrations of 5-FU induced ICD, accompanied by the release of tumor-associated antigens and damage-associated molecular patterns. Simultaneously, R837 activated dendritic cells and promoted macrophage polarization toward the M1 phenotype, synergistically enhancing antitumor immunity.

This probiotic-guide platform, which functions as an orally administered *in situ* vaccine, represents a safe and effective strategy for CRC chemo-immunotherapy. The combination of nanotechnology and probiotic-mediated delivery provides a robust foundation for the development of precision oral drug delivery systems, with potential applications in the treatment of various intestinal diseases.

## CRediT authorship contribution statement

**Wenfei Fan:** Data curation, Formal analysis, Investigation, Methodology, Writing – original draft, Writing – review & editing. **Shuaiguang Li:** Data curation, Formal analysis, Funding acquisition, Investigation, Supervision, Validation, Writing – original draft, Writing – review & editing. **Xue Wang:** Data curation, Formal analysis, Investigation, Methodology, Writing – review & editing. **Jingya Xu:** Data curation, Formal analysis, Investigation, Supervision, Writing – review & editing. **Shan Jiang:** Data curation, Formal analysis, Supervision, Writing – review & editing. **Haixia Shen:** Data curation, Formal analysis, Investigation, Methodology, Writing – review & editing. **Yanran Yue:** Data curation, Formal analysis, Investigation, Methodology, Writing – review & editing. **Zhonghua Dong:** Data curation, Formal analysis, Supervision, Validation, Writing – review & editing. **Xuan Wang:** Data curation, Formal analysis, Investigation, Validation, Writing – review & editing. **Haiping Hu:** Formal analysis, Methodology, Writing – review & editing. **Wei Xu:** Conceptualization, Formal analysis, Funding acquisition, Project administration, Resources, Writing – original draft, Writing – review & editing.

## Declaration of competing interest

The authors declare that they have no known competing financial interests or personal relationships that could have appeared to influence the work reported in this paper.

## Data Availability

Data will be made available on request.
