# Supplementary material for: An orally administered biohybrid complex leveraging probiotic guidance to treat colorectal cancer via in situ vaccination
Source: Mater Today Bio. 2026 Jul 14;39:103445. doi: 10.1016/j.mtbio.2026.103445 (PMC13400377; doi:10.1016/j.mtbio.2026.103445)
Supplement: Multimedia component 1 [file mmc1.docx]

**Supporting Information**

An orally administered biohybrid complex leveraging probiotic guidance to treat colorectal cancer via *in situ* vaccination

*Wenfei Fan ^a,†^, Shuaiguang Li ^a,b,c,†^, Xue Wang ^a^, Jingya Xu ^a^, Shan Jiang ^a,c^, Haixia Shen ^d^, Yanran, Yue ^a,c^, Zhonghua Dong ^a^, Xuan Wang ^a^, Haiping Hu ^e^, Wei Xu ^a,*^*

*^a^ Department of Clinical Pharmacy, The First Affiliated Hospital of Shandong First Medical University & Shandong Provincial Qianfoshan Hospital, Jinan, Shandong 250014, China*

*^b^ State Key Laboratory of Advanced Drug Delivery and Release Systems, Shandong Luye Pharmaceutical Co., Ltd., Yantai, Shandong 264003, China.*

*^c^ School of Pharmaceutical Sciences, Shandong University of Traditional Chinese Medicine, Jinan, Shandong 250355, China*

*^d^ School of Pharmacy, The Shandong Second Medical University, Weifang, Shandong 261053, China*

*^e^ Department of Pharmacy, The Second Affiliated Hospital of Shandong First Medical*

*University, Tai'an, Shandong 271000, China.*

*Corresponding author. Tel.: +86 17686791319;

Correspondence to: Prof. Wei Xu, E-mail: [weixu@sdu.edu.cn](mailto:weixu@sdu.edu.cn); [wxu@email.sdfmu.edu.cn](mailto:wxu@email.sdfmu.edu.cn).

Wenfei. Fan and Shuaiguang. Li contributed equally to this work.

**Experimental section**

**1. Penetration effect of CS@CB-Lipo@5-FU/R837 on tumor spheroids.**

Prior to seeding, the wells were pre-coated with a 3D cell coating solution to prevent cell adhesion to the well walls and thereby promote efficient spheroid formation. After gently mixing the cell suspension to ensure homogeneity, 100 μL CT26 of the cell suspension (2×10^3^ cells) was aliquoted into each well of a Thermo Fisher 96-well U-bottom ultra-low attachment plate using a multichannel pipette. The plate was gently tapped on its sides to settle the cells to the center of the well bottom. The plate was then carefully transferred to a 37 °C, 5% CO_2_ incubator. Movement of the plate was avoided for the initial 24 h to prevent disruption of spheroid formation. The spheroids were cultured for an additional 3 days until they reached the desired size and exhibited compact structures; this time point was designated as day 0 for subsequent experiments.

During subsequent experimental procedures, most of the spent medium was carefully aspirated from each well (leaving a small volume to avoid exposing and damaging the spheroids), and replaced with 100 μL of drug-containing media from different experimental groups (free 5-FU, Lipo@5-FU/R837, and CS@Lipo@5-FU/R837). Morphological changes of the tumor spheroids were observed and recorded under an inverted microscope on days 1, 3, and 5.

**2. Western blot analysis of ICD-related proteins**

Total cellular proteins were extracted from CT26 cells after 24 h of treatment with different formulations. Protein concentrations were determined using a BCA protein assay kit. Equal amounts of protein (20 μg per lane) were separated by 10% SDS-PAGE and subsequently transferred onto PVDF membranes. The membranes were blocked with 5% non-fat milk in TBST (Tris-buffered saline containing 0.1% Tween-20) for 2 h at room temperature, followed by overnight incubation at 4 °C with primary antibodies against CRT (1:1000), HMGB1 (1:1000), and GAPDH (1:10000), respectively. After three washes with TBST, the membranes were incubated with HRP-conjugated secondary antibodies (1:50000) for 2 h at room temperature. Protein bands were visualized using an enhanced chemiluminescence (ECL) substrate and imaged with a chemiluminescence imaging system. Semi-quantitative analysis of band intensities was performed using ImageJ software, with GAPDH serving as the loading control.

**Supplementary Figures**

**Figure S1.** UV–vis spectra of CB, CB-N_3_(PEG_4_) and CB-N_3_(PEG_2000_).

**Figure S2.** Relative quantitative analysis of mean fluorescence intensity (MFI) of fluorescent gel image of AF488-labeled CB, CB-N_3_ (PEG_4_) and CB-N_3_(PEG_2000_). Data are presented as the mean ± SEM. Statistical significance was determined using one-way ANOVA with Tukey’s multiple comparisons. ***P*  <  0.01, *****P*  <  0.0001.

**Figure S3.** (A) CLSM images of Cy5 labelled-N_3_ of CB-N_3_(PEG_2000_). (B) Flow cytometry analysis of DBCO-Cy5 of CB and CB-N_3_(PEG_2000_), and (C) quantitative analysis of (MFI). Data are presented as the mean ± SEM. Statistical significance was determined using one-way ANOVA with Tukey’s multiple comparisons. *****P*  <  0.0001.

**Figure S4.** Agar plates of CB (PEG_4_) and CB-N_3_(PEG_2000_).

**Figure S5.** (A) Colony formation assay of CB, CB-Lipo@5-FU/R837, and CS@CB-Lipo@5-FU/R837, and (B) colony counting. (C) OD value of 600 nm. Data are presented as the mean ± SEM. Statistical significance was determined using one-way ANOVA with Tukey’s multiple comparisons. ns, *P* > 0.05, **P*  <  0.05.

**Figure S6.** Serum stability of Lipo@5-FU/R837 *in vitro*. (A) Representative TEM image of Lipo@5-FU/R837 after incubation in serum. Scale bar: 1 μm. (B) Particle size and (C) zeta potential changes of Lipo@5-FU/R837 in serum over 8 h. Data are presented as the mean ± SEM. Statistical significance was determined using one-way ANOVA with Tukey’s multiple comparisons. ns, *P* > 0.05.

**Figure S7.** Serum stability of CS@CB-Lipo@5-FU/R837 *in vitro*. (A) Representative TEM image of CS@CB-Lipo@5-FU/R837 after incubation in serum. Scale bar: 500 nm. (B) Particle size and (C) zeta potential changes of CS@CB-Lipo@5-FU/R837 in serum over 8 h. Data are presented as the mean ± SEM. Statistical significance was determined using one-way ANOVA with Tukey’s multiple comparisons. ns, *P* > 0.05.

**Figure S8.** (A) DC activation and (B) quantitative statistics of different proportions of drugs (5-FU or R837). Data are presented as the mean ± SEM. Statistical significance was determined using one-way ANOVA with Tukey’s multiple comparisons. ***P*  <  0.01, ****P*  <  0.001, *****P*  <  0.0001.

**Figure S9.** (A) Cell viability of L929 cells under different treatments for 24 h and 48 h using CCK-8 kit. (B) Live/dead staining of MC38 cells. Green fluorescence indicates Calcein-AM staining for living cells, and red indicates propidium iodide (PI) staining for dead cells. Data are presented as the mean ± SEM.

**Figure S10.** Uptake of AF488-labeled liposomes by CT26 cells. (A) CLSM images of cellular uptake of AF488-labeled liposomes, (B) quantitative analysis of fluorescence intensity, and (C-D) flow cytometry results. Data are presented as the mean ± SEM. Statistical significance was determined using one-way ANOVA with Tukey’s multiple comparisons. ***P*  <  0.01, ****P*  <  0.001.

**Figure S11.** (A) Time-dependent colocalization images of CS@Lipo@AF488 with lysosomes for 1 h, (B) 2 h and (C) 4 h, respectively.

**Figure S12.** (A) Representative luminescence images of tumor spheroid size using CT26-luc cells, and (B) and quantitative luminescence analysis. Data are presented as the mean ± SEM. Statistical significance was determined using one-way ANOVA with Tukey’s multiple comparisons. *****P*  <  0.0001.

**Figure S13.** (A) Cytokine levels of IL-10, (B) IL-6, (C) IL-12, and (D) TNF-α in supernatants. Data represent the mean ± SEM. Statistical significance was determined using one-way ANOVA with Tukey’s multiple comparisons. ns, *P* > 0.05, ***P*  <  0.01, ****P*  <  0.001, *****P*  <  0.0001.

**Figure S14.** ATP release in supernatants, normalized to the PBS group. Data represent the mean ± SEM. Statistical significance was determined using one-way ANOVA with Tukey’s multiple comparisons. ns, *P* > 0.05, ***P*  <  0.01, ****P*  <  0.001, *****P*  <  0.0001.

**Figure S15.** Western blot of HMGB1 and CRT in different groups. GAPDH was used as internal control.

**Figure S16.** Relative protein levels of HMGB1 (A) and CRT (B) in different groups (n=6). Data represent the mean ± SEM. Statistical significance was determined using one-way ANOVA with Tukey’s multiple comparisons. **P*  <  0.05, ***P*  <  0.01, ****P*  <  0.001, *****P*  <  0.0001.

**Figure S17.** (A)*Ex vivo* fluorescence imaging of intestinal distribution of free DiR, CB-Lipo@DiR and CS@CB-Lipo@DiR in mice. (B) After oral administration of CS@CB-Lipo@5-FU/R837 with identical initial bacterial dosage, normal colon mucosa (b) and tumor tissues (c) were homogenized, serially diluted and cultured on selective agar plates. Under *in vitro* culture conditions, the number of viable bacteria in free CB (a) was consistent with that in CS@CB-Lipo@5-FU/R837 (b, c). Data represent the mean ± SEM. Statistical significance was determined using one-way ANOVA with Tukey’s multiple comparisons. ns, *P* > 0.05, *****P*  <  0.0001.

**Figure S18.** Agar plate images of tumor tissue homogenates under different formulations.

**Figure S19.** Tumor size in orthotopic CRC models after treatment (n = 6).

**Figure S20.** Spleen size (A) and weight (B) in orthotopic CRC models after treatment (n = 6). Data are presented as the mean ± SEM. Statistical significance was determined using one-way ANOVA with Tukey’s multiple comparisons. ns, *P* > 0.05, **P*  <  0.05, ****P*  <  0.001, *****P*  <  0.0001.

**Figure S21.** Representative images of H&E, Ki67, TUNEL, CRT and HMGB1 staining of tumor tissues.

**Figure S22.** Tumor growth during treatment in subcutaneous CRC models (n = 6).

**Figure S23.** Relative IOD values of CD4^+^ T cells (A) and CD8^+^ T cells (B) detected by immunohistochemistry in different groups of subcutaneous CRC models after treatment. Data represent the mean ± SEM. Statistical significance was determined using one-way ANOVA with Tukey’s multiple comparisons. **P*  <  0.05, ***P*  <  0.01, ****P*  <  0.001, *****P*  <  0.0001.

**Figure S24.** H&E staining images of organs in each group after treatment in orthotopic CRC models.

**Figure S25**. H&E staining images of organs in each group after treatment in subcutaneous CRC models.

**Figure S26.** (A) Biochemical parameters of white blood cell (WBC), (B) red blood cell (RBC), (C) platelet in blood (PLT), (D) alanine aminotransferase (ALT), (E) aspartate aminotransferase (AST), and (F) creatinine (CREA). Data are presented as the mean ± SEM.

**Figure S27.** Gating strategy. (A) Gating strategy for identifying CD8⁺ T cells and CD4⁺ T cells in tumor tissues. (B) Gating strategy for defining CD86⁺ and CD206⁺ cell populations in tumor tissues.

**Figure S28.** Quantification of CD8⁺ T cell infiltration (n = 4). Data are presented as the mean ± SEM. Statistical significance was determined using one-way ANOVA with Tukey’s multiple comparisons. ns, *P* > 0.05, **P*  <  0.05, ****P*  <  0.001, *****P*  <  0.0001.

**Figure S29.** Analysis of immune status in tumor tissue. (A) Expression of CD44 and CD62 in CD8⁺T cells. (B) Statistical analysis of the proportion of CD44⁺CD62L⁻ subset in CD8⁺T cells. (n = 4). Data represent the mean ± SEM. Data were analyzed using one-way ANOVA with Tukey's post hoc test. ns, *P* > 0.05, ^*^*P* < 0.05, ^**^*P* < 0.01.

**Figure S30.** Statistical analysis of the proportion of CD206⁺ subset in F4/80⁺ cells (n = 4). Data are presented as the mean ± SEM. Statistical significance was determined using one-way ANOVA with Tukey’s multiple comparisons. ns, *P* > 0.05, **P*  <  0.05.

**Figure S31.** Gating strategy. (A) Gating strategy for identifying CD8⁺ T cells, CD4⁺ T cells, and Foxp3⁺ expression within CD4⁺ T cells in the spleen. (B) Gating strategy for detecting CD86⁺ and CD80⁺ cells in the spleen. (C) Gating strategy for analyzing the intracellular levels of TNF-α and IFN-γ in CD8⁺ T cells from tumor tissues.

**Figure S32.** Analysis of immune status in tumor tissue. (A) Representative FCM analysis of DC maturation and (B) percentage of Foxp3⁺ cells among CD4⁺ T cells in orthotopic CRC models after treatment.

**Figure S33.** DC maturation (A) and Foxp3⁺ Treg proportions in CD4⁺ T cells (B) in orthotopic CRC models after treatment (n = 4). Data are presented as the mean ± SEM. Statistical significance was determined using one-way ANOVA with Tukey’s multiple comparisons. ns, *P* > 0.05, **P*  <  0.05, ***P*  <  0.01, ****P*  <  0.001, *****P*  <  0.0001.

**Figure S34.** Analysis of immune status in spleen. (A) Expression of CD4⁺ T cells and CD8⁺ T cells in CD45⁺ cells. (B) Expression of CD8^+^ T cells in CD45^+^CD3^+^CD8^+^ cells. (C)The ratios of CD8/CD4. (D) Proportion of CD8⁺ T cells/Treg ratios quantification (n = 4). Data are presented as the mean ± SEM. Statistical significance was determined using one-way ANOVA with Tukey’s multiple comparisons. ns, *P* > 0.05, **P*  <  0.05, ***P*  <  0.01, ****P*  <  0.001.

**Figure S35.** (A) Cytokine levels of IFN-γ and (B) TNF-α measured by FCM. (C) Levels of GZMB, (D) TNF-α, (E) IL-12, (F) IL-6, and (G) IL-10 detected by ELISA. Data are presented as the mean ± SEM. Statistical significance was determined using one-way ANOVA with Tukey’s multiple comparisons. ns, *P* > 0.05, **P*  <  0.05, ***P*  <  0.01, ****P*  <  0.001, *****P*  <  0.0001.

**Figure S36.** Immunohistochemical staining of CD69 and CD103 in orthotopic CRC tumor tissues after different treatments (n = 3). Scale bar: 100 μm.

**Figure S37.** Quantitative analysis of CD69^+^ (A) and CD103^+^ (B) positive area percentages in orthotopic CRC tumor tissues after different treatments. Data are presented as the mean ± SEM. Statistical significance was determined using one-way ANOVA with Tukey’s multiple comparisons. **P*  <  0.05, ***P*  <  0.01, *****P*  <  0.0001.

**Figure S38.** Tumor growth in the rechallenge model. (A) Representative images of the re-challenged tumors after different treatments. (B) Tumor weights of the rechallenged tumors (n = 6). Data are presented as the mean ± SEM. Statistical significance was determined using one-way ANOVA with Tukey’s multiple comparisons. *****P*  <  0.0001.

**Figure S39.** Immune status analysis in tumor tissues of the rechallenge model. (A) Representative flow cytometry analysis of CD8⁺ T cells in tumor tissues, (B) quantitative analysis of CD8⁺ T cells and (C) quantitative analysis of CD8⁺/CD4⁺ ratios. Data are presented as the mean ± SEM. Statistical significance was determined using one-way ANOVA with Tukey’s multiple comparisons. **P*  <  0.05. ***P*  <  0.01.

**Figure S40.** Immune status analysis in tumor tissues of the rechallenge model. (A) Representative FCM plots of DC maturation and (B) quantitative analysis. Data are presented as the mean ± SEM. Statistical significance was determined using one-way ANOVA with Tukey’s multiple comparisons. *****P*  <  0.0001.

**Figure S41.** Immune status analysis in tumor tissues of the rechallenge model. (A, C) Representative FCM plots of macrophage polarization, and (B, D) quantitative analysis. Data are presented as the mean ± SEM. Statistical significance was determined using one-way ANOVA with Tukey’s multiple comparisons. **P*  <  0.05. *****P*  <  0.0001.

**Figure S42.** Immune status analysis in tumor tissues of the rechallenge model. (A) Percentage of Foxp3⁺ cells among CD4⁺ T cells, and (J) quantitative analysis of Foxp3⁺ cells among CD4⁺ T cells. Data are presented as the mean ± SEM. Statistical significance was determined using one-way ANOVA with Tukey’s multiple comparisons. ****P*  <  0.001.

**Figure S43.** Cytokines in the tumor rechallenge model. (A) Representative FCM plots of GZMB. (B) Quantitative analysis of GZMB proportions in CD8⁺ T cells. Data are presented as the mean ± SEM. Statistical significance was determined using one-way ANOVA with Tukey’s multiple comparisons. ***P*  <  0.01.

**Figure S44.** Cytokines in the tumor rechallenge model. (A) Representative FCM plots of IFN-γ. (B) Quantitative analysis of IFN-γ proportions in CD8⁺ T cells. Data are presented as the mean ± SEM. Statistical significance was determined using one-way ANOVA with Tukey’s multiple comparisons. **P*  <  0.05.

**Figure S45.** Cytokines in the tumor rechallenge model. (A) Representative FCM plots of TNF-α. (B) Quantitative analysis of TNF-α proportions in CD8⁺ T cells. Data are presented as the mean ± SEM. Statistical significance was determined using one-way ANOVA with Tukey’s multiple comparisons. **P*  <  0.05.

**Figure S46.** Alterations in gut microbiota composition. (A) *Firmicutes*/*Bacteroidetes* (F/B) ratios at the phylum level. (B) Relative abundance of the orders *Lachnospirales* and *Bacteroidales*. (C) Principal coordinate analysis (PCoA) plot illustrating beta diversity differences among groups (n = 3). Data are presented as the mean ± SEM. Statistical significance was determined using one-way ANOVA with Tukey’s multiple comparisons. **P*  <  0.05.

**Supplementary Tables**

**Table S1** Pharmacokinetic parameter of various formulations (n = 3).

| **Formulation** | ***C_max_*_(5-FU)_(µg/mL)** | ***T_max_*(h)** | **AUC_0-4_(µg*h/mL)** | **F_rel_%** |
| --- | --- | --- | --- | --- |
| 5-FU | 0.500 ± 0.027 | 0.5 | 1.144 ± 0.029 | 100 |
| Lipo@5-FU | 1.120 ± 0.059 | 2.0 | 3.034 ± 0.109 | 265 |
| CB-Lipo@5-FU | 1.238 ± 0.052 | 2.0 | 3.503 ± 0.176 | 306 |
| CS@CB-Lipo@5-FU | 1.632 ± 0.114 | 2.0 | 4.449 ± 0.115 | 389 |

**Table S2** Antibody information used in the experiment.

|  | **Antibody** | **Dilution** | **Item number** | **Company** |
| --- | --- | --- | --- | --- |
| 1 | PE-Cyanine 7 anti-mouse CD11b | 1:400 | Cat# 101216 | BioLegend, Inc., USA |
| 2 | Brilliant Violet 605 anti-mouse CD86 | 1:200 | Cat# 105037 | BioLegend, Inc., USA |
| 3 | APC anti-mouse CD206 | 1:400 | Cat# 141708 | BioLegend, Inc., USA |
| 4 | PE anti-mouse CD80 | 1:200 | Cat# 104707 | BioLegend, Inc., USA |
| 5 | PE Cyanine 7 anti-mouse CD8 | 1:200 | Cat# 100722 | BioLegend, Inc., USA |
| 6 | FITC anti-mouse CD11c | 1:400 | Cat# 117305 | BioLegend, Inc., USA |
| 7 | Brilliant Violet 421 anti-mouse F4/80 | 1:200 | Cat# 123131 | BioLegend, Inc., USA |
| 8 | Brilliant Violet 510 anti-mouse IA/IE | 1:400 | Cat# 107636 | BioLegend, Inc., USA |
| 9 | Percp-Cyanine 5 anti-mouse CD4 | 1:400 | Cat# 100434 | BioLegend, Inc., USA |
| 10 | Brilliant Violet 510 anti-mouse CD44 | 1:400 | Cat# 103044 | BioLegend, Inc., USA |
|  | **Antibody** | **Dilution** | **Item number** | **Company** |
| 11 | FITC anti-mouse Foxp3 | 1:400 | Cat# 126406 | BioLegend, Inc., USA |
| 12 | Brilliant Ultra Violet 395 anti-mouse CD4 | 1:400 | Cat# 563790 | BD Biosciences, USA |
| 13 | Brilliant Violet 650 anti-mouse CD8 | 1:200 | Cat# 563234 | BD Biosciences, USA |
| 14 | APC-Cyanine 7 anti-mouse CD8 | 1:200 | Cat# 561967 | BD Biosciences, USA |
| 15 | Biotin anti-mouse CD11c | 1:400 | Cat# 568970 | BD Biosciences, USA |
| 16 | Biotin anti-mouse CD11b | 1:400 | Cat# 553309 | BD Biosciences, USA |
| 17 | Biotin anti-mouse NK-1 | 1:400 | Cat# 569231 | BD Biosciences, USA |
| 18 | Biotin anti-mouse Gr-1 | 1:400 | Cat# 553327 | BD Biosciences, USA |
| 19 | Biotin anti-mouse Ter-119 | 1:400 | Cat# 553672 | BD Biosciences, USA |
| 20 | Percp-Cyanine 5.5 anti-mouse SA | 1:400 | Cat# 561560 | BD Biosciences, USA |
| 21 | Brilliant Violet 650 anti-mouse CD62L | 1:400 | Cat# 564108 | BD Biosciences, USA |
| 22 | Brilliant Violet 786 anti-mouse CD45 | 1:400 | Cat# 564225 | BD Biosciences, USA |
| 23 | Brilliant Ultra Violet 737 anti-mouse CD3e | 1:200 | Cat# 612771 | BD Biosciences, USA |
| 24 | Fixable Viability Stain 700 | 1:2000 | Cat# 564997 | BD Biosciences, USA |
| 25 | Fixation/Permeabilization Concentrate | 1:200 | Cat# 00-5123-43 | Thermo Fisher Scientific, Inc. |
| 26 | Foxp3 Permeabilization Buffer | 1:200 | Cat# 00-5523-00 | Thermo Fisher Scientific, Inc. |
|  | **Antibody** | **Dilution** | **Item number** | **Company** |
| 27 | Permeabilization Buffer | 1:200 | Cat# 88-8824-00 | Thermo Fisher Scientific, Inc. |
| 28 | Brefeldin A | 1:200 | Cat# 00-4506-51 | Thermo Fisher Scientific, Inc. |
